# Signalling couples hair follicle stem cell quiescence with reduced histone H3 K4/K9/K27me3 for proper tissue homeostasis

**DOI:** 10.1038/ncomms11278

**Published:** 2016-04-15

**Authors:** Jayhun Lee, Sangjo Kang, Karin C. Lilja, Keegan J. Colletier, Cornelia Johanna Franziska Scheitz, Ying V. Zhang, Tudorita Tumbar

**Affiliations:** 1 Department of Molecular Biology and Genetics, Cornell University, Ithaca, New York 14853, USA

## Abstract

Mechanisms of plasticity to acquire different cell fates are critical for adult stem cell (SC) potential, yet are poorly understood. Reduced global histone methylation is an epigenetic state known to mediate plasticity in cultured embryonic SCs and T-cell progenitors. Here we find histone H3 K4/K9/K27me3 levels actively reduced in adult mouse skin and hair follicle stem cells (HFSCs) during G0 quiescence. The level of marks over specific gene promoters did not correlate to mRNA level changes in quiescent HFSCs. Skin hypomethylation during quiescence was necessary for subsequent progression of hair homeostasis (cycle). Inhibiting BMP signal, a known HFSC anti-proliferative factor, elevated HFSC methylation *in vivo* during quiescence prior to proliferation onset. Furthermore, removal of proliferation factors and addition of BMP4 reduced histone methylases and increased demethylases mRNAs in cultured skin epithelial cells. We conclude that signalling couples hair follicle stem cell quiescence with reduced H3 K4/K9/K27me3 levels for proper tissue homeostasis.

## Abstract (web summary)

Changes in global histone trimethylation have been linked to embryonic but not adult stem cell plasticity. Here, Lee *et al*. find H3 K4/K9/K27me3 levels actively reduced in adult mouse skin and hair follicle stem cells during quiescence (catagen) and link this to active bone morphogen protein signalling.

## Graphical abstract

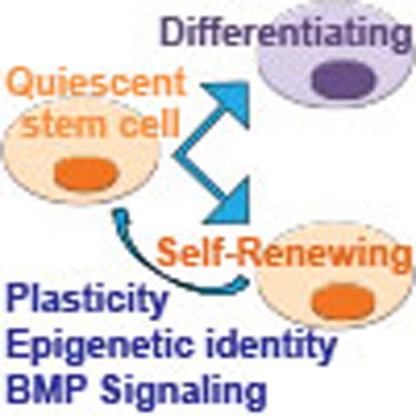

Plasticity can be defined as the ability of cells to maintain a flexible genome and adopt various cell fates, and has been previously linked with specialized chromatin states in pluripotent stem cells (SCs)^1^,^2^,^3^,^4^,^5^. Adult tissue stem cells (TSCs) are rare and difficult to access in their intact niche in a living organism, and the link between chromatin states and TSC plasticity to make differentiated cells during adult tissue homeostasis is poorly understood^6^. Recently, work in the adult hair follicle stem cells (HFSCs) suggested that the plasticity to adapt to different environments such as the natural niche, the injured skin or cultured conditions *in vitro* is influenced by pioneer factors that couple their activity with open chromatin domains known as super-enhancers^7^. However, how super-enhancers or other epigenetic states influence the intrinsic plasticity of adult HFSCs during normal homeostasis, as a fundamental mechanism that allow these cells to both self-renew and also adopt differentiated hair lineage fates remains unclear. Interestingly, embryonic stem cell (ESC) plasticity of fate determination is characterized by decreased ‘epigenomic identity', defined as low global levels of several histone epigenetic marks such as H3K9me3 and H3K27me3 (refs 2, 3, 4, 5; hypomethylation). Genetic interference with the level of such epigenetic marks affected ESCs pluripotency^8^,^9^ as well as the ability of committed cells to dedifferentiate and be reprogrammed^1^,^10^. Similarly, T-cell progenitors in culture showed hypomethylation of H3 K27/K9me3, and this was confined to their G0 quiescent state, when cells do not divide. Interestingly, this hypomethylation conferred T-cell progenitors elevated genome plasticity during quiescence, to be more efficiently reprogrammed to pluripotency^11^. Finally, muscle SCs *in vivo* also showed hypomethylation of H3K27me3 in quiescence, but a correlation with plasticity was not yet made^12^. It is unclear if histone H3 hypomethylation of specific lysine residues is generally associated with quiescence of tissue stem and progenitor cells, and if there is any functional significance *in vivo* for this specific state. Also unclear is how such global, apparently genome-wide, reduction in histone methylation might relate to specific levels of gene expression. This is important because many, although not all, adult TSCs reside in G0 quiescence in their niche for long time periods, and upon activation are expected to rapidly adapt and perform tissue regeneration^13^.

Mouse HFSCs are an excellent model system for studying the link between SC plasticity and quiescence because they undergo synchronous phases of quiescence and proliferation that are tightly regulated. Furthermore, hair follicles have defined morphology, which permits unambiguous *in situ* identification of the SCs (bulge, Bu region), progenitor cells (matrix, Mx region) and differentiated lineages (inner layers, ILs; Fig. 1a). Furthermore, bulge HFSCs can be freshly sorted from skin in large quantities based on cell surface markers CD34 and α6-integrin, and this allows biochemical analyses, which are inaccessible to many other tissue systems. Hair homeostasis occurs in regenerative cycles (Fig. 1a,b) composed of successive synchronous stages of tissue remodelling: telogen (rest and quiescence of HFSCs), anagen (proliferation/differentiation of matrix progenitors and independent self-renewal of Bu HFSCs) and catagen (regression/apoptosis of progenitor and differentiated lineages, re-structuring of the SC niche and quiescence of the HFSCs)^14^,^15^.

As HFSCs enter quiescence at late anagen/early catagen, they are re-positioned relative to the residence niche (bulge); eventually their ultimate position, by the end of catagen, dictates their subsequent fate (Fig. 1b). In one fate, the HFSCs would become early progenitor hair germ cells, destined to divide rapidly at anagen, differentiate to matrix and further to terminally differentiated lineages in the ILs, and eventually die. In the second fate, the HFSCs would self-renew symmetrically within the bulge by undergoing several (∼3 ×) rounds of cell division during early anagen, become crowded in their niche and eventually cease proliferation to enter quiescence again in late anagen^16^,^17^,^18^,^19^. It follows that at catagen, quiescent HFSCs have higher cell fate potential or plasticity (two possible fates: differentiate/die or self-renew) when compared with proliferative HFSCs at anagen (single fate: self-renewal; Fig. 1b)^20^,^21^.

Here we examine global levels and genome distribution of histone H3 trimethylation marks commonly associated with transcriptional repression (H3K9me3 and H3K27me3) and activation (H3K4me3)^22^ in HFSCs (of CD34+ bulge SCs) during self-renewal (early anagen) and quiescence (catagen). We show for the first time that quiescent HFSCs present global hypomethylation of specific H3 marks, relative to their proliferative counterparts. Hypomethylation of HFSCs peaks at catagen (before fate decisions), and was not previously reported by *Lien et al*.^23^, who focused on different hair cycle stages. Moreover, we demonstrate that methylation levels are actively controlled by diffusible proliferation inhibitory signals (bone morphogenetic protein (BMP)) but are not immediately relevant to mRNA levels in quiescent HFSCs. Importantly, hypomethylation throughout the skin at catagen appears crucial for subsequent normal hair cycle progression.

## Results

### Reduction of H3 K4/K9/K27me3 in quiescent HFSCs

**Figure 1:**
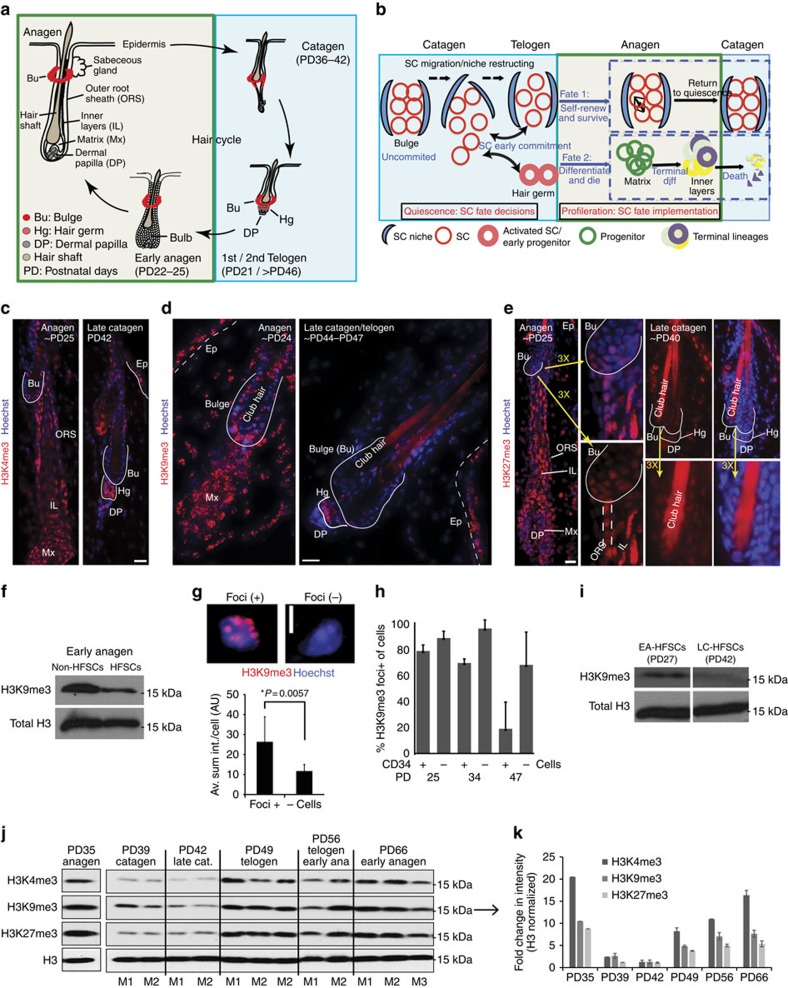
Distinct low levels of histone H3 K4/K9/K27me3 in quiescent (catagen) HFSCs. (**a**,**b**) Depiction of hair cycle and fate choices of bulge HFSCs during quiescence, before activation. (**c**,**e**) Immunofluorescence staining of histone methyl marks at hair cycle stages indicated (Bu, bulge; DP, dermal papillae; Ep, epidermis; Hg, hair germ; IL, inner layers; Mx, matrix; ORS, outer root sheath). Scale bars, 10 μm. (**f**) Western blot of H3K9me3 in histone extracts from FACS-isolated cells. (**g**) Quantification of fluorescence intensity of *z*-stack images of Foci+ and Foci- cells in the bulge from PD24 hair follicle sections shows a significant difference (Student's t-test). AU, Arbitrary Unit. Scale bar, 5 μm. (**h**) Fraction of H3K9me3 foci+ cells, quantified on cytospin of FACS-isolated cells derived from mice at PD25 (anagen, proliferation), PD34 (late anagen, ceasing proliferation) and PD47 (telogen, quiescence). (**i**) Western blot of H3K9me3 on FACS-isolated HFSCs at early anagen (EA) and late catagen (LC). Notice the reduction of H3K9me3 in late catagen. (**j**) Western blot of histone marks on total skin histone extracts from different stages of hair cycle. Notice the reduction of all three marks in catagen skin relative to other stages. All western blots showed a single band between 15 and 17 kDa region, which corresponds to the correct size of histone H3 protein. (**k**) Band intensities from **j** were quantified and normalized to total H3 to calculate the fold differences. Fold changes were averaged among mice at the same stage.

To probe the levels of H3K4me3, H3K27me3 and H3K9me3 in HFSCs relative to more differentiated cells and at different stages of homeostasis, we examined mouse skin sections at different postnatal days (PDs) by immunofluorescence. We found that at anagen, bulge cells (HFSCs) were generally less stained than lower outer root sheath (ORS), progenitor (matrix) cells and differentiated hair lineages (ILs). The epidermis also generally showed higher methylation levels (Fig. 1c–e and Supplementary Fig. 1a,b). To rule out differences in antibody penetration in different cell types and validate our results of immunofluorescence data, we sorted HFSCs (bulge cells as CD34+/α6-integrin+) and non-HFSCs (mostly epidermis and hair follicle lower ORS cells as CD34−/α6-integrin+) at early anagen (∼PD25) by FACS (fluorescence-activated cell sorting) and performed a test western blot analysis for one of our marks relative to total histone H3 (Fig. 1f). This confirmed global reduction of H3K9me3/H3 in HFSCs relative to non-HFSCs and demonstrated that our immunofluorescence methods are good reporters of overall global levels of methylation. Furthermore, bulge HFSCs showed strikingly lower (but not absent) immunofluorescence signal levels of histone marks during quiescence (Late Catagen, LC-HFSCs) relative to proliferative (Early Anagen, EA-HFSCs; Fig. 1c–e, and Supplementary Fig. 1a–c). Because histone H3K9me3 shows distinct bright nuclear foci, we could readily quantify the distribution of foci-positive and -negative cells within the CD34+ bulge HFSCs at different hair cycle stages (Fig. 1g,h), which confirmed lowest level for catagen bulge cells. The late catagen stage was assessed based on morphology, and was also confirmed using Caspase-3 staining (Supplementary Fig. 1d,e). To verify that antibody immunofluorescence staining correctly report on levels of histone methylation in bulge cells at catagen, we performed western blots of sorted CD34+/α6-integrin+ HFSCs at early anagen (PD25) and late catagen (∼PD42-44) using histone H3K9me3/H3 as test of principle (Fig. 1i). This validated our immunofluorescence methods for different hair cycle stages, and verified hypomethylation of bulge HFSCs at late catagen. Immunofluorescence suggested a most pronounced reduction of methylation in the bulge HFSCs relative to other skin compartments, but tissue sections at catagen consistently appeared less stained throughout the skin. This observation was confirmed by western blots of total skin tissue and quantification for all the H3 trimethylation marks relative to histone H3 (Fig. 1j,k). These data together demonstrated that catagen is a stage when skin is generally hypomethylated for H3 K4/K9/K27me3. Furthermore, bulge HFSCs show hypomethylation at catagen (in quiescence) relative to early anagen (proliferation) and also relative to its more differentiated hair lineages (matrix and IL) at anagen.

**Figure 2:**
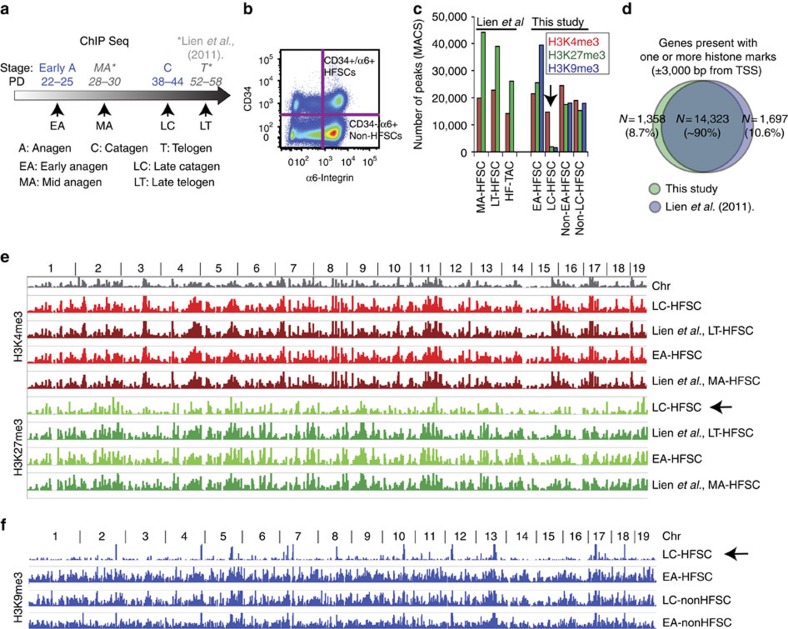
ChIP-seq of HFSCs reveals genome-wide reduction of H3 K9/K27me3 in catagen. (**a**) Scheme of hair cycle stages that were used for ChIP-seq of this work (blue) and Lien *et al*. (* gray)^23^. (**b**) Representative FACS plot that shows the sorting scheme of HFSCs and non-HFSCs. (**c**) Total number of peaks called for all three marks using MACS^24^. Raw ChIP-seq signals from publically available databases (GEO accession: GSE31239) were employed in our analysis methods to generate peaks for the Lien *et al*.^23^ study that were directly comparable with our data. Note the reduction in the total peak number in LC-HFSCs (arrow), especially prominent for K9/K27me3. (**d**) Venn diagram of all genes present with one or more histone marks in their transcription start sites, within 3,000 bp upstream and downstream. Note that ∼90% of genes are commonly marked in the two studies that used independent ChIP-seq methods. (**e**) Genome view of MACS peaks from ChIP-seq data of this study and of the study by Lien *et al*.^23^. Chr, chromosome number. Note that large-scale enrichment patterns across the genome are similar between the two studies, as expected. H3K27me3 shows visible reduction in overall signal in LC-HFSCs. (**f**) Genome-wide view of H3K9me3 (K9) ChIP-seq peaks in different sorted populations. Chr, chromosome number. Notice a dramatic reduction in the overall signal in LC-HFSCs.

To examine genome-wide distribution and levels of DNA-bound methylated histone H3 K4/K9/K27me3, we performed chromatin immunoprecipitation followed by sequencing (ChIP-seq). We used comparable number of sorted CD34+/α6-integrin+ EA-HFSCs (PD22–25) and LC-HFSCs (PD38–44; Fig. 2a,b). We also sorted and analysed non-HFSCs (nEA and nLC) counterparts as CD34−/α6-integrin+. Using IgG as background for normalization, our data revealed good correlation between experiments and enrichments in specific functional genomic regions, such as promoters, introns and repetitive regions, as expected (Supplementary Fig. 2a,b). We compared our data with a previous ChIP-seq study of H3K4me3 and H3K27me3 (H3K9me3 was not analysed) by *Lien et al*.^23^, done at Mid/late Anagen (MA-HFSCs) and Late Telogen (LT-HFSCs; Fig. 2a–e). The overall genome-wide distribution for the marks was similar in the two studies (Fig. 2d,e), however, our data uniquely revealed fewer ChIP-seq peaks in LC-HFSCs when compared with EA-HFSCs (Fig. 2c and Supplementary Fig. 2c). Close examination of ChIP-seq data distribution across the genome showed that for H3K27me3 and especially for H3K9me3 not only the total number of peaks but also the height was dramatically decreased in LC-HFSCs (Fig. 2e,f, arrows). In case of H3K4me3, the number of peaks were marginally decreased in LC-HFSCs relative to the other populations (Fig. 2c,e), which prompted us to examine the ChIP-seq signals in a manner that would allow more quantitative examination of signal at discrete chromosomal loci.

**Figure 3:**
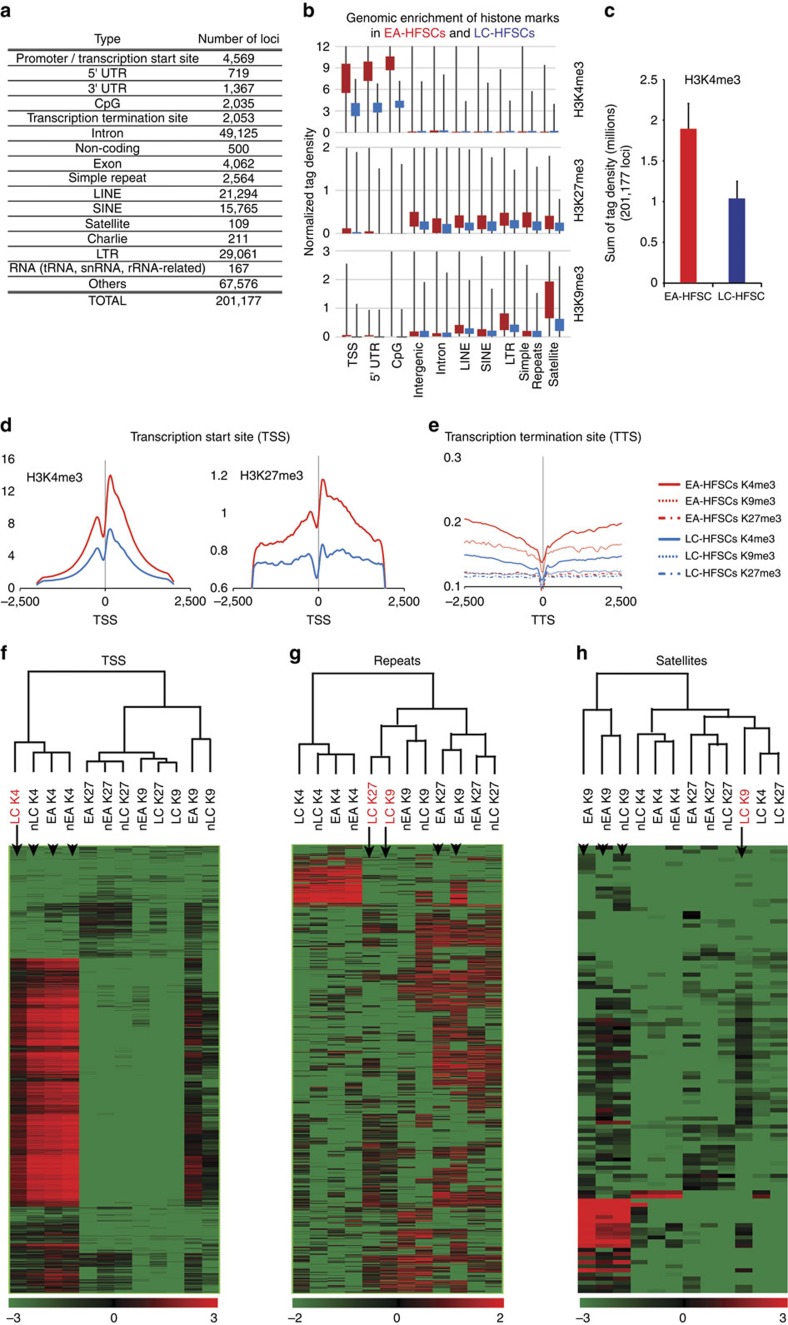
Reduction of H3 K4/K9/K27me3 at functional genomic regions at catagen. (**a**) List of all genomic regions covered by MACS peaks from three marks in all four populations. (**b**) Box whisker plot of genomic enrichment of histone marks in EA-HFSCs (red) and LC-HFSCs (blue). Number of reads was normalized to 1 million of total mapped reads (normalized tag density). Note that the individual histone marks are higher at expected regulatory sites relative to the rest of the genome (for example, enrichment of H3K4me3 in TSS/promoters, H3K27me3 in intergenic and H3K9me3 in satellite regions), yet display overall decrease compared with EA-HFSCs. See the Methods for detail. (**c**) Average sum of all tag densities of H3K4me3 in all chromosomal loci analysed. Note that the overall peak intensity is lower by ∼2-fold in LC-HFSCs. (**d**) Average distribution of H3K4me3 (left panel) and H3K27me3 (right panel) in transcription start sites (TSS) of EA-HFSCs (red) and LC-HFSCs (blue) and (**e**) all three marks in transcription termination sites (TTS; right panel). Note the clear reduction of marks in LC-HFSCs. (**f**–**h**) Quantitative heat plot analysis of H3K4me3 (K4), H3K27me3 (K27) and H3K9me3 (K9) levels on TSS (**f**), simple repeats (**g**) or satellite regions (**h**). Notice the reduction of marks in LC-HFSCs highlighted in red (arrow versus arrowheads). UTR, untranslated region; LINE, Long Interspersed Nuclear Element; SINE: Short Interspersed Elements; LTR, Long Terminal Repeats.

For this, we adapted an existing sequencing data analysis pipeline that allows direct comparisons of fold changes at defined chromosomal loci across samples (see the Methods for details). We defined 201,177 chromosomal loci (Fig. 3a), in which at least one of the three marks was detected as a ‘peak' (via MACS, model-based analysis of ChIP-Seq^24^) in any one of our populations. We then quantified the signal as ‘normalized tag density' (number of sequencing reads mapped within a specific region per total number of mapped reads normalized across analysed samples; see the Methods for details). As expected for these marks, this analysis revealed enrichment of H3K4me3 at transcription start sites (TSS), 5′ untranslated region and CpG sites, whereas H3K27me3 and H3K9me3 were enriched in regions elsewhere, such as repetitive satellite regions (Fig. 3b).

In all regions analysed, the signal appeared higher in EA-HFSCs than LC-HFSCs. This was true even for the H3K4me3 signal, which was indeed decreased not only at TSS but also overall in LC-HFSC relative to EA-HFSC by ∼2-fold (Fig. 3c). The decrease was in line with our previous observations, but the fold change was less than expected from our western blots and immunofluorescence data. One possible explanation for this apparent difference, aside from inherent caveats of different methods of comparisons, is that in addition to its enrichment within defined narrow peaks analysed in ChIP-seq, H3K4me3 mark might also have a broad distribution across the genome, which is detected as ‘background' and hence lost in peak calling methods. Examination of H3K4me3 levels distribution across transcription start (TSS) and termination sites, also revealed consistent elevated levels for EA-HFSCs relative to LC-HFSCs (Fig. 3d,e and Supplementary Fig. 2d). Similarly, H3K27me3 and H3K9me3 were also decreased in LC-HFSCs across several types of chromosomal loci analysed (Fig. 3b,e). In addition, a quantitative approach using FPKM (Fragments Per Kilobase of transcripts per Million mapped reads^25^) signals allowed clustering analysis and heat mapping of ChIP-seq signals, which confirmed once more hypomethylation of specific chromosomal loci in LC-HFSCs for all the marks (Fig. 3f–h). We conclude that LC-HFSCs show global hypomethylation of H3 K4/K9/K27me3 relative to EA-HFSCs, and that this is true for chromatin-bound H3 histones at specific and functional genome-wide elements.

### Poor mRNA and H3 K4/K9/K27me3 correlation in quiescence

**Figure 4:**
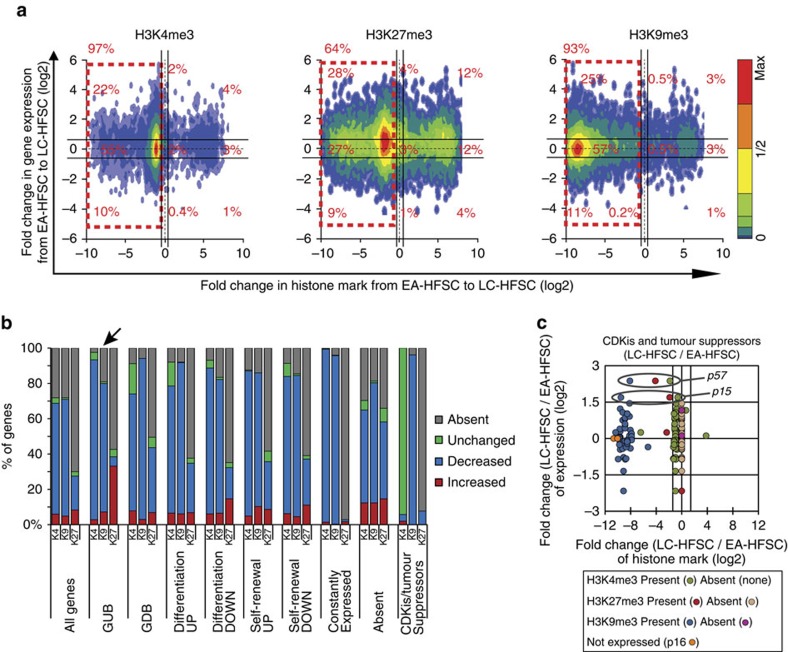
Correlation of level changes in H3 K4/K9/K27me3 changes and mRNA. (**a**) Density plot of all genes in the mouse genome that show signal by either microarray or ChIP-seq (*N*=24,778 genes and *N*=10,345 genes plotted for H3K4me3/H3K9me3 and H3K27me3, respectively, out of total *N*=34,497 genes. Genes that showed the absence of microarray or ChIP signals in comparing populations were excluded). Genes changed ≥1.5-fold are marked outside the black lines flanking the origin; genes with <1.5-fold variations are considered ‘unchanged'. (**b**) % of genes that are decreased (blue), unchanged (green) and increased (red) in their histone marks from EA-HFSCs to LC-HFSCs. Each functional category was defined based on changes in mRNA levels in HFSCs at distinct stages of differentiation and self-renewal (GDB: genes downregulated in the bulge). See the Methods for description of arrays used and Supplementary Data 2 for the lists of genes. Note atypical behaviour of genes upregulated in the bulge (GUB) and tumour suppressors. (**c**) Dot plot of cyclin-dependent kinase inhibitors (CDKis) and known tumour suppressors that play a role in skin and hair follicle biology (Supplementary Data 2). Expression of CDKis was adopted from our previous qRT–PCR data^46^, whereas mRNA values for the other genes were from microarrays (Supplementary Data 1).

The global hypomethylation observed in LC-HFSCs prompted us to question the correlation between changes in levels of mRNAs and H3 K4/K9/K27me3 levels at specific TSS gene loci. We used our Affymetrix microarray data from sorted HFSCs (Supplementary Data 1, see the Methods for details) and selected 24,778 genes (for H3 K4/K9me3) and 10,345 genes (for H3K27me3) that showed detectable signal in either microarray or ChIP-seq analysis. We found that in LC-HFSCs, irrespective of changes in mRNA levels, ∼90% genes with H3K4me3 or H3K9me3 on TSS and 64% with H3K27me3 on TSS downregulated the mark (Fig. 4a). As with the analysis of the whole genome, this was again most pronounced for H3K9me3 and least pronounced for H3K4me3. Our analysis of data from the study by Lien *et al*.^23^ showed similar reduction for H3K27me3 but not for H3K4me3 in LT-HFSCs (Supplementary Fig. 3); it is likely that H3K4me3 was already regained by the late telogen stage analysed in that study, as also suggested by our western blot analysis (Fig. 1j). Also note that normalization to IgG (not available for Lien *et al*.^23^) accounts for accessibility of antibodies to the chromatin and noticeably modify quantitative ChIP-seq data distribution (Supplementary Fig. 3). Comparison of EA-HFSCs and non-EA-HFSCs showed better correlation of mRNA and histone mark levels (Supplementary Fig. 3b), suggesting that the LC-HFSCs might represent a special case (see the Discussion for details).

**Figure 5:**
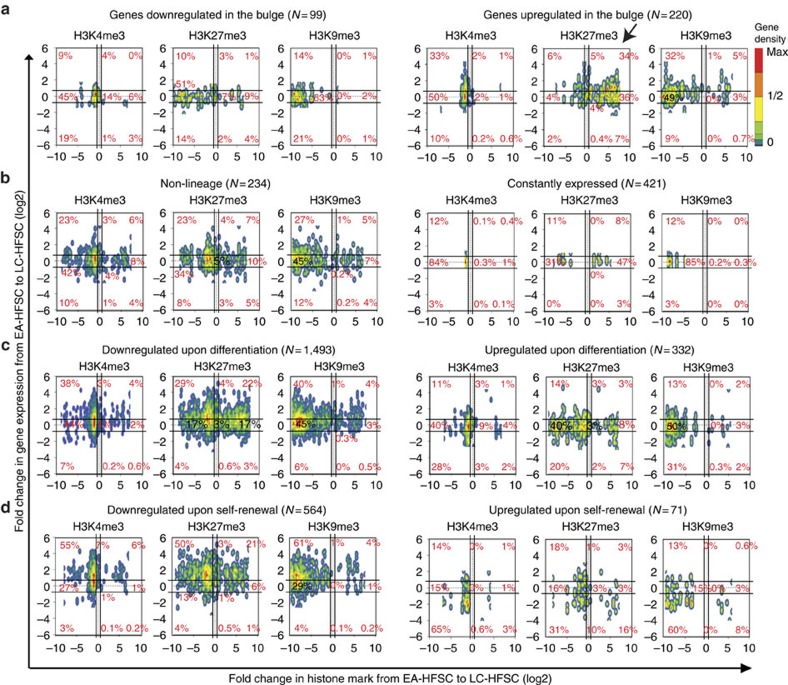
Correlation of histone marks and gene expression between EA- and LC-HFSCs. (**a**–**d**) PD25 (EA-HFSCs, self-renewing stage) and PD42 (LC-HFSCs, prior to fate decisions) expression arrays were compared with identified groups of genes that behave similarly in their expression across populations and hair cycle stages (see the Methods for acquisition of array and ChIP-seq signals). Fold changes between EA-HFSCs and LC-HFSCs (LC-HFSCs/EA-HFSCs) in array and ChIP-seq signals were log2 transformed, followed by conversion of data matrix into a density plot using R. Genes that were absent with array or ChIP-seq signals were omitted in the plot. Majority of the gene sets analysed showed reduction of H3K27me3 and H3K9me3, regardless of their expression patterns. H3K4me3 levels are relatively less dramatically changed. Interestingly, GUB uniquely show increased level of H3K27me3 from anagen bulge to catagen bulge, suggesting they are under special regulation. These data suggest that the change in gene expression as defined by mRNA levels does not necessarily correlate with the change in histone modification levels. Refer to Supplementary Data 2 for a complete list of ChIP and array signals.

Next, we asked if subsets of genes that we pre-define based on function, might be differentially methylated at the HFSCs transition to quiescence. General histone H3 K4/K9/K27me3 decrease at TSS in LC-HFSCs relative to EA-HFSCs was detectable in all functionally defined gene sets analysed (Figs 4b and 5a–d, and Supplementary Data 2), with two notable exceptions. First, a relatively large fraction of Genes Upregulated at the mRNA level at all hair cycle stages in the Bulge (GUB) showed elevated H3K27me3 mark in LC-HFSCs (Figs 4b and 5a, arrow), whereas all other gene sets showed reduced H3K27me3 levels. Second, a set of genes previously defined as cell cycle regulators and skin tumour suppressors did not show a decrease of H3K4me3 level at the TSS in LC-HFSCs, like all the other gene sets did, and displayed a striking and atypical lack of H3K27me3 in both LC-HFSCs and EA-HFSCs (Fig. 4b,c, and Supplementary Data 2). These data show that most TSSs lose their histone marks to some degree in LC-HFSCs, and this is irrespective of the mRNA level changes. This loss is most pronounced for H3K9me3, and least pronounced for H3K4me3, whereas H3K27me3 show intermediate decrease and special behaviour at specific gene sets, suggesting these are under specific regulation. These data suggest that the level of H3 K4/K9/K27me3 does not dictate mRNA levels in quiescent HFSCs at catagen.

### H3 K4/K9/K27me3 reduction in quiescence governs homeostasis

**Figure 6:**
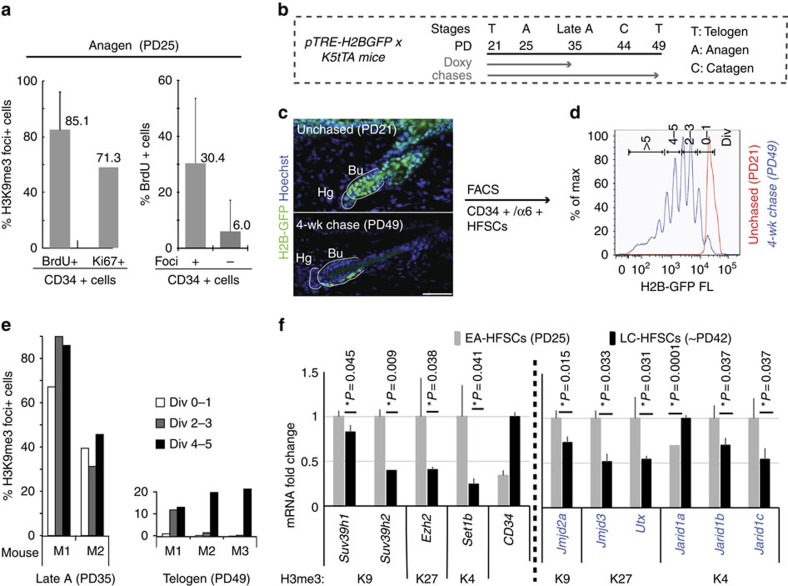
Potential regulation of histone methylation state by histone-modifying enzymes. (**a**) Quantification of foci + and − in CD34+/BrdU+ cells and CD34+/Ki67^+^ cells (left panel), and % BrdU+ cells in CD34+/foci+ and CD34+/foci− cells. (**b**) H2B-GFP pulse-chase scheme to examine histone mark level relative to HFSC divisional history. (**c**) Skin sections and (**d**) FACS plots of PD21 (unchased) and PD21–49 doxy chased mice. H2B-GFP FACS signal dilutes ½ upon division. Scale bars, 50 μm. (**e**) % H3K9me3 foci+ in cytospin collection of FACS-isolated HFSCs with different divisional history from late anagen (left) and telogen (right) mice. (**f**) qRT–PCR of multiple H3 methylases and demethylases *in vivo.* Statistical significance was analysed using Student's *t*-test. Refer to Supplementary Table 1 for list of primers used. wk, week.

To examine how histone methyl mark levels are regulated, we considered three possibilities: (i) direct correlation with cell cycle entry; (ii) passive dilution over multiple divisions and (iii) changes in levels of histone-modifying enzymes^26^. We examined correlation with cell cycle entry by quantifying H3K9me3 immunofluorescence signal as foci-positive and -negative cells in CD34+ bulge cells co-stained for proliferation markers 5-bromodeoxyuridine (BrdU) or Ki67 at anagen (PD25). We found that the majority (>70%) of BrdU+ and Ki67+ HFSCs at anagen were H3K9me3 foci+ (Fig. 6a, left), suggesting that actively cycling HFSCs are generally highly methylated. The un-methylated CD34+ bulge cells (H3K9me3 foci−) showed only 6% proliferative (BrdU+) cells, whereas methylated CD34+ cells showed 30% BrdU+ cells (Fig. 6a, right). These data suggested that the high levels of marks and active cell cycle are somewhat, but not strictly, correlated in CD34+ bulge cells at anagen. In fact, at late anagen (PD34) when HFSCs are already known to have exited cells cycle and entered G0 quiescence^16^, the level of marks appeared still high (Fig. 1h and Supplementary Fig. 1). All these data suggested that H3K9me3 levels did not strictly correlate with cell cycle and were likely related to hair cycle stage.

Simultaneously, we had examined if hypomethylation was a result of marks being passively diluted over divisions that occurred in the bulge at anagen. We counted H3K9me3 foci-positive and -negative cells in CD34+/α6+ HFSCs that were FACS separated based on their division history over one hair cycle. Divisions were indicated by H2B-GFP dilution in 2- and 4-week doxycycline chases performed on *pTRE-H2BGFP; K5tTA* mice^18^,^19^ (Fig. 6b–d, see the Methods for details). All cells irrespective of division history showed relatively high levels of marks at late anagen (PD35). By PD49, all bulge cells showed relative loss of marks, but the frequently divided bulge cells showed slightly more marks than the infrequently divided bulge cells (Fig. 6e). These results contradict the passive dilution model.

Finally, we examined mRNA levels by quantitative real-time reverse transcriptase–PCR (qRT–PCR) of FACS-isolated (CD34+/α6+) LC-HFSCs (∼PD42) and EA-HFSCs (∼PD25), focusing on candidate chromatin-related genes identified by previous microarray data^16^,^27^ (Fig. 6f and Supplementary Fig. 4a). Importantly, we found that H3 methylases *Ezh2* (H3K27me3), *Suv39h1/2* (H3K9me3) and *Set1b* (H3K4me3) were highly expressed in EA-HFSCs and were significantly downregulated in LC-HFSCs (Fig. 6f, left). This was in line with our observed high level of histone marks in EA-HFSCs and the decreased level in LC-HFSCs (Figs 1, 2, 3). A number of histone demethylases for each mark were also expressed in EA-HFSCs (Fig. 6f, right) and these could be responsible for the decrease in marks we detected by late catagen. Most of these genes (with the exception of *Jarid1a*) were also downregulated by catagen (Fig. 6f, right), which might indicate that LC-HFSCs reached a steady state that does not require maintenance of high levels of histone methylases or demethylases.

**Figure 7:**
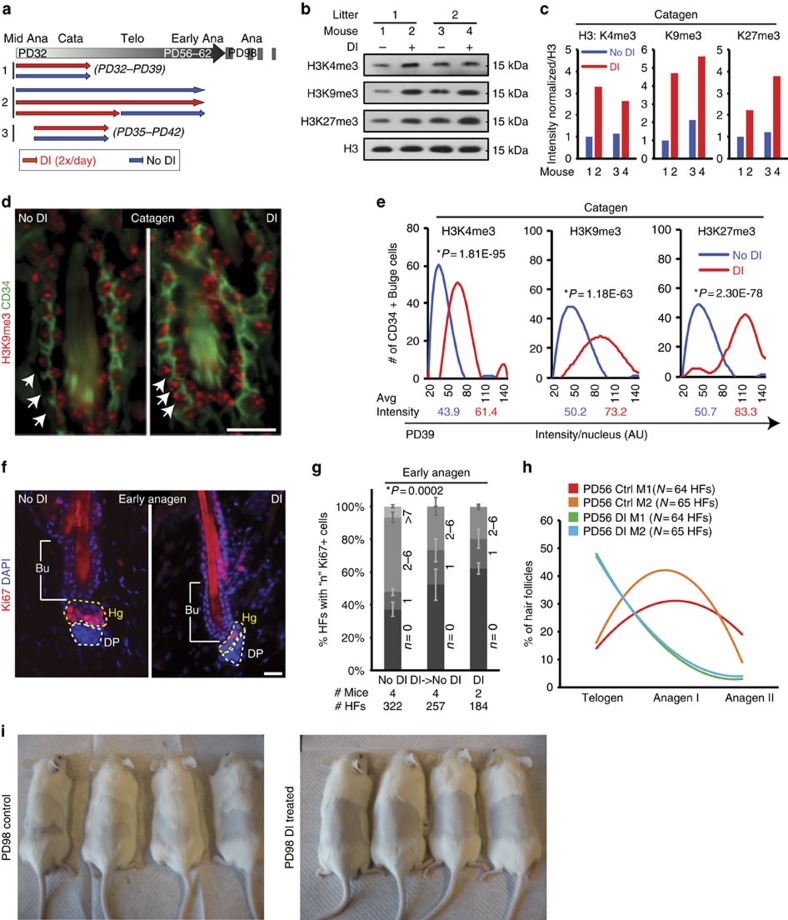
The level of histone H3 K4/K9/K27me3 in the skin is important for hair homeostasis. (**a**) Experimental scheme of using demethylase inhibitors (DIs). (**b**,**c**) Western blot (**b**) and quantification of band intensity (**c**) of histone methylation from whole skin chromatin extracts of control and DI-treated mice. Band intensities were normalized to H3 signal. (**d**) Immunofluorescence staining of control and DI-treated (scheme 1 of (**a**)) hair follicle sections labelled with H3K9me3 (red) and CD34 (green). Scale bar, 20 μm. (**e**) Fluorescence intensity of all three marks from immunostainings were quantified for each bulge cell (CD34+). Student's *t*-test was used to determine statistical significance between control and DI-treated mice for each mark. (**f**) Immunofluorescence staining with a proliferation marker Ki67 of control and DI-treated (scheme 2 of **a**) skin sections. Scale bar, 20 μm. (**g**) The number of Ki67^+^ cells in **f** in each hair follicle were counted, and they showed reduced proliferation in DI-treated mice compared with control. The differences across the conditions were significant according to a Student's *t*-test. (**h**) Hair follicles of control and DI-treated mice were categorized in their hair cycle stages. Notice a dramatic increase in the number of telogen (Telo) hair follicles and reduction in anagen (Ana) I and II in DI-treated mice. (**i**) Mice were shaved, treated with DI in catagen (Cata; PD35–42; **a**, scheme 3), and followed long-term to monitor the hair cycle re-entry. Notice that mice in the control group show hair growth, whereas mice in DI-treated group show no growth. Avg, average.

Next, we examined the potential implication of histone demethylases in erasure of histone H3 K4/K9/K27me3 marks in HFSCs at catagen, and probed the functional significance of these levels. We employed demethylase chemical inhibitors (DIs) known to be specific for H3 K4me3, K9me3 (ref. 28) and K27me3 (ref. 29), shown by biochemical and structural studies to have potent and specific activities towards these modifications. We applied a cocktail of DI onto mouse back skin from anagen (PD32) up to different time points, and assessed the proliferation status of hair follicle in the subsequent hair cycle stage at early anagen (Fig. 7a). We used mice from a 1:1 mixed (CD1 × Fvb) genetic background, which we previously found to have a relatively shorter second telogen and more consistent early onset of the second adult anagen^27^,^30^, than other common mouse strains. Western blot analysis of histones extracted from total mouse skin DI-treated for 7 days (PD32–39; Fig. 7a, scheme 1) showed increased histone methylation levels (Fig. 7b,c) confirming the activity of DI *in vivo*. Similarly, CD34+ bulge cells also showed increased methylation levels by immunofluorescence and its quantification (Fig. 7d,e). When we interrogated timing of early anagen onset at the expected later stages (∼PD56–62), the mice treated with DI (Fig. 7a, scheme 2), showed a delay relative to untreated (no DI) mice both by morphology and by number of proliferative hair germ cells (Fig. 7f–h). Finally, mice treated with DI in catagen (PD35–42; Fig. 7a, scheme 3) failed to grow new hair in the subsequent hair cycle when monitored long-term up to PD98, whereas control mice clearly showed hair shaft growth throughout substantial portions of the shaved region (Fig. 7i). Taken together, these results demonstrate the importance of reducing H3 K4/K9/K27 trimethylation levels in the skin during quiescence for proper subsequent hair follicle homeostasis.

### BMP signalling regulates H3 K4/K9/K27me3 levels

**Figure 8:**
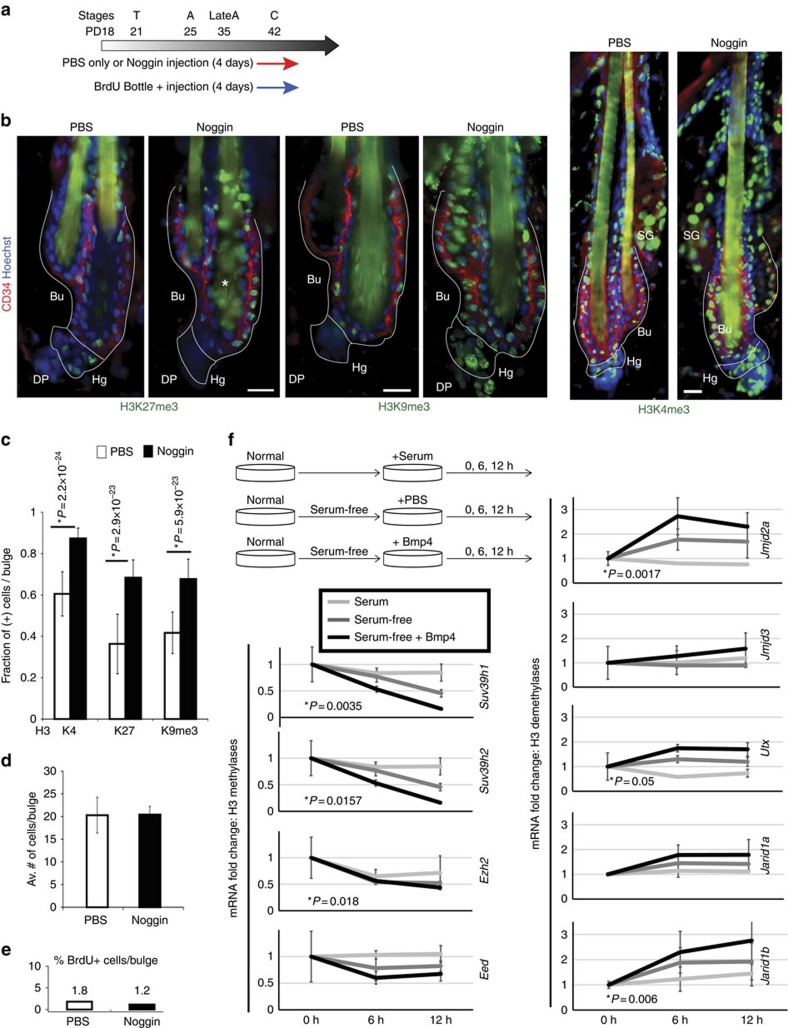
BMP signalling is involved in control of the global histone marks level. (**a**) Noggin- or PBS-coated fluorescent microbeads were intradermally injected for 4 days in late catagen. BrdU was fed to mice in the water bottles to continuously label proliferating cells. (**b**) Representative images of immunofluorescence skin staining showing increased of methylation marks in Noggin-injected mice. Scale bars, 10 μm. (**c**) Quantification of methylation signal in bulge cells show a significant increase in cells positively bearing histone marks in Noggin-injected (black bar) mice relative to PBS control (white bar) mice (*n*=2 mice, *N*>40 hair follicles per condition, Student's *t*-test). (**d**,**e**) Quantification of number of CD34+ cells per bulge (**d**) and BrdU+ cells per bulge (**e**) after 4 days of injection shows no significant differences between Noggin and PBS control, suggesting lack of proliferation of bulge cells. *N*=2 mice per condition, *n*=20–40 hair follicles per mouse. (**f**) qRT–PCR of selected histone methylases and demethylases in keratinocytes cultured under serum (white line), serum-free (grey line) or serum-free+BMP4 (black line) conditions. Note the reduced expression of methylases and increase of demethylases upon BMP4 addition. Statistical significance was determined by Student's *t*-test. Results for all genes tested are shown in Supplementary Fig. 4b.

Because the decreased levels of methylation observed to occur by late catagen was apparent in the whole skin (Fig. 1c–g and Supplementary Fig. 1a,b), we wondered if a skin diffusible factor might be implicated in regulation. Skin at catagen/telogen lacks diffusible proliferation signals, such as epidermal growth factor (EGF) and Wnt, and elevates inhibitory signals, such as BMP^14^. Indeed, we find that intra-dermal injection of Noggin (Fig. 8a), a BMP antagonist^31^, during the long second telogen was sufficient to increase the histone H3 K4/K9/K27me3 mark levels in bulge cells (Fig. 8b,c) before activation of proliferation (Fig. 8d,e). Furthermore, withdrawal of serum (lack of proliferative signals) and addition of BMP4 (increase of repressive signal) in cultured keratinocytes downregulated a number of methylases and upregulated demethylases at their mRNA level (Fig. 8f and Supplementary Fig. 4b). Histone methylation levels did not change noticeably upon serum removal and addition of BMP4 during the 12-h course of this experiment, as judged by microscopy of immunofluorescence-labelled cells, suggesting demethylation might be a relatively slow process. Alternatively, *in vitro* conditions might not completely mimic the skin environment at catagen onset. Nevertheless, these results demonstrate that BMP signalling, known to induce and maintain HFSC quiescence *in vivo*^14^,^31^ can also produce immediate changes in mRNA levels of methylases and demethylases in skin keratinocytes. These changes are consistent with an eventual decrease of methylation, which is detectable *in vivo* at the time of hair cycle when the BMP signals are known to be elevated and begin to act^14^,^31^. Moreover, we found that bulge HFSCs depend on active BMP signals *in vivo* to maintain low levels of histone H3 methylation.

## Discussion

**Figure 9:**
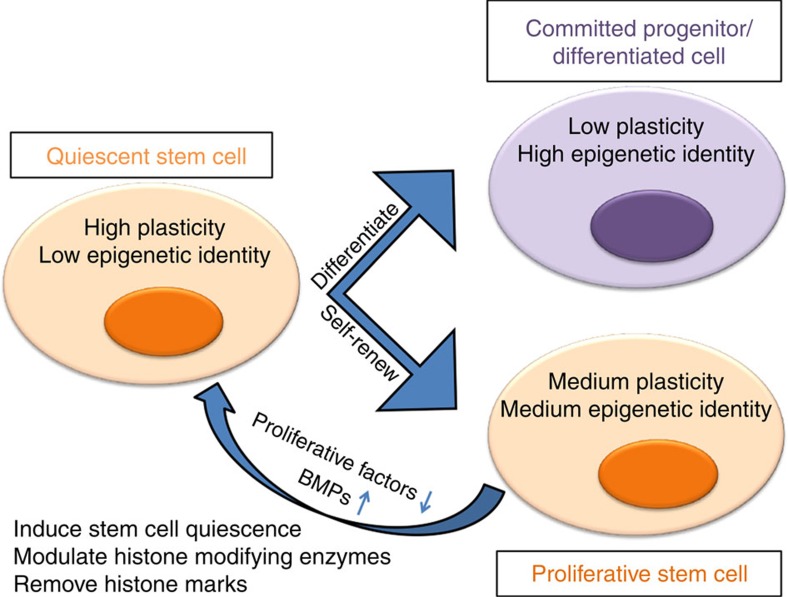
Model for signalling coupled regulation of chromatin state and genome plasticity. Hair follicles undergo dramatic remodelling at catagen as they prepare for new growth in the next hair cycle. Hair follicle stem cells at this stage are faced with a bidirectional cell fate choice to (i) remain as a stem cell in the bulge and self-renew at anagen or (ii) migrate out from their niche before division and eventually become a progenitor matrix cell that fuel the hair growth^16^,^20^,^21^. Signalling (that is, BMP) that plays a crucial role in regulating quiescence of hair follicle stem cells^14^, also may control the level of multiple histone-modifying enzymes, leading to a global reduction of several key histone modification marks previously implicated in transcriptional activation or repression. Such global reduction in histone marks may place the quiescent hair follicle stem cell epigenome at a ‘blank' (or a plastic) state. This would subsequently allow efficient re-writing of the methylation marks throughout the genome in the pattern required to enforce the ultimate cell fate choice. The latter is dictated by the ultimate position within the niche in which the quiescent stem cell finds itself at the onset of hair growth.

Our data suggest that quiescent HFSCs display chromatin features previously associated with high plasticity and low epigenetic identity in ESCs and in quiescent T-cell progenitors^2^,^11^. These are characterized by low levels of histone H3 K4/K9/K27me3 in HFSCs at catagen. Suggestively, this state occurs at the stage during hair homeostasis when quiescent HFSCs are most naïve with respect to fate choice and are preparing for subsequent decisions to either differentiate or self-renew (Fig. 9). Strikingly, the global decrease in levels of histone H3 K4/K9/K27me3 we found at this stage did not affect the mRNA levels in the expected direction. One possible explanation is that steady-state mRNA levels detectable by microarrays are not an accurate reflection of active transcription, and that at catagen mRNA levels are determined by post-transcriptional regulation. Another possibility is that in quiescence (in the absence of cell division) methylation level does not have a direct impact on ongoing or active transcription. Methylation impact on transcription can occur not only by change in binding of transcription factors at functional elements, but also at the large-scale chromatin level. For instance, physical association *in trans* of genes with large heterochromatic structures (associated with high level of H3K9me3) could broadly enforce gene repression^11^,^32^. Genome-wide changes in histone methylation levels suggest that regulation at the large-scale chromatin level may be present in the hair follicle homeostasis.

Histone methylation has been deemed a long-range type of epigenetic mark that enforces patterns of gene expression over *multiple rounds of cell division*^33^. HFSCs do raise their H3 K4/K9/K27me3 levels back up at late telogen/early anagen, before onset of multiple rounds of imminent cell divisions. We propose methylation might be needed only in dividing cells to enforce inheritance of gene expression patterns both in the differentiating rapidly dividing matrix cells, as well as in dividing bulge cells. This way, the bulge-specific SC patterns of gene expression must be transmitted over the multiple (1–5x) rounds of symmetric bulge-cell divisions that replenish the SC pool^16^,^30^. During anagen, HFSCs remain confined to the bulge and do not differentiate to matrix or IL until they finish self-renewal, exit the cell cycle and pass once again through a new stage of G0 quiescence at catagen/telogen^16^. During this quiescence stage, we showed that the histone methyl marks are erased, perhaps reducing the strong identity of these cells as SCs. This ‘erasure' could create an ‘empty page' in the genome, onto which new marks could later on be rapidly and efficiently added in the required patterns to either make matrix (progenitors) or bulge (stem) cells later on when proliferation starts again. The pattern corresponding to make either one of the two possible fates (matrix versus bulge) would be dictated by the location of the HFSCs within the niche (bulge) or outside the niche (hair germ). This model would predict that maintaining the levels of histone methylation high throughout quiescence in SCs, would block them from adopting a normal hair germ fate, characterized by abilities for subsequent rapid activation and proliferation at anagen onset. As recent studies have shown, HFSCs possess heterogeneous behaviour with respect to their spatial positioning within the niche^34^. Thus, in the future, additional markers that would allow one to more finely tease apart different populations of HFSCs (that is, old bulge versus new bulge or upper bulge versus lower bulge) as well as the ability to sort SCs during early catagen (as they are migrating outside of their niche and are not yet established as new bulge versus hair germ cells), may be utilized to more directly probe the regulation of histone methylation in HFSC fate plasticity. With that said, before anagen onset it seems both hair germ cells and bulge cells retain the ability to interconvert^16^,^19^,^34^,^35^,^36^, thus their plasticity likely remains high until proliferation onset, which might irreversibly seal their fate.

Because of multiple methylases and demethylases that are simultaneously expressed in the bulge, genetic targeting to simultaneously modulate H3 K4/K9/K27me3 levels in HFSCs is not trivial. Our chemical inhibition of three classes of demethylases during 1-week of catagen in which subsequent hair growth is extensively delayed, comes close in supporting our model, although it cannot exclude the possible effects from the environment. Further testing will be needed in future by creating epithelial-conditional mouse models with concomitant forced-expression of several methylases in each class and/or concomitant knockdown of multiple demethylases, to complement our chemical inhibition studies. The advent of new genetic targeting approaches^37^ might make this endeavour feasible in the foreseeable future. Suggestively, overexpression *of Ezh2* in haematopoietic SCs promoted self-renewal at the expense of differentiation^38^, supporting our model. Importantly, the onset and maintenance of both quiescence^14^,^31^ and low histone marks (this work) coincide and seem to be regulated via the same signals (BMP) that temporally and functionally couple these two processes in the tissue. Recent studies concur with our model, and suggest that BMP signalling greatly influences the outcome of bulge SC fate^39^, and pSmad1/5, a BMP effector, directly binds upstream of multiple histone demethylases in HFSCs from second telogen^40^. However, ChIP-seq of pSmad1/5 at catagen is not currently available, and it remains possible that the regulation of H3 K4/K9/K27me3 levels throughout the skin by BMP occurs indirectly, by secondary induction of an intermediate pathway.

That demethylation observed at catagen occurs throughout the skin and is not epithelial specific is interesting, and is possible that BMP works in parallel with other signals at this stage to influence levels of methylation in different skin compartments. This is especially relevant as nuclear pSmad1/5 is only detected in the hair follicle but not in the epidermis^40^. In cell culture, loss of proliferative signals influence methylase/demethylase mRNA expression in a manner similar to the addition of BMP (this work). Thus, it is possible that lack of proliferative (that is, Wnt) signals *in vivo*, might influence the level of methylation in different skin compartments, such as epidermis, at catagen. Indeed, recent studies suggest that β-catenin is highly present in the basal layer of interfollicular epidermis and modulates transcription of demethylases^41^, some of which are crucial for epidermal self-renewal^42^. Finally, in HFSCs, the Wnt pathway effector TCF3/4 directly binds to promoters of demethylases, suggesting direct transcriptional regulation^41^. Thus, the coupling between signalling, histone-modifying enzymes and methylation levels in different skin compartments, as well as their overall impact in skin biology might involve complex signalling networks that require further investigation.

Finally, it will be interesting to examine how the level of histone H3 methylation marks might interface with other mechanisms of HFSC plasticity. Recently, it has been shown that HFSCs are characterized by the activity of a number of super-enhancers that have been defined as chromatin domains with high levels of H3 K27 acetylation, low levels of H3K27me3 and the clustering of mediator together with specific HFSC transcription factors^7^. These domains are remodelled during differentiation as well as in injury and culturing conditions, but it is unclear what their status might be during catagen, when global hypomethylation is instated in HFSCs, and how this status might compare with self-renewing HFSCs during early anagen when methylation levels are globally high.

In conclusion, our work suggests an intriguing model in which tissue SC quiescence and genome plasticity are linked by signals that temporally couple tissue homeostasis with histone H3 K4/K9/K27me3 hypomethylation. Our model might be relevant to other tissue SC systems. Many, although not all, tissue SCs are preferentially quiescent and/or undergo dynamic stochastic or environmentally driven choices of either differentiation or self-renewal^20^,^43^. The most potent haematopoietic SCs reside in G0 quiescence and divide only five times in a lifetime^13^. Notably, quiescent muscle SCs^12^ and quiescent progenitor T cells also displayed globally hypo-methylated histones, and this state enhanced reprogramming efficiency in cultured T cells^11^. Thus, quiescence and plasticity, two fundamental characteristics of many tissue SCs, might be coupled via homeostatic signals and specialized chromatin states in tissues beyond hair follicles. It will be interesting to explore if hypomethylation plays a role in plasticity of not only quiescent but also more actively cycling tissue SCs, such as those of the intestine. These SCs might resemble more the situation recently described for proliferating progenitor cells in *Drosophila* embryos, where genes that change expression dramatically during development lack methylation marks^44^. Perhaps hypomethylation may be differentially implemented in distinct contexts to promote similar outcomes of enhancing plasticity for proper cell fate determination.

## Methods

### Mice, growth factor-beads injections

All mice were treated according to Cornell University Institutional Animal Care and Use Committee protocols. We used littermates CD1 × FVB wild-type strain for our analysis including histone methylation characterization and ChIP-seq. Both males and females were used for most analysis and control and experimental groups were matched by sex. For delivery of growth factor-coated beads, we used an approach previously described^18^,^31^,^45^. A small region of the mouse back skin was shaved. Recombinant mouse Noggin (R&D Systems) was injected intradermally (0.5–5 μg ml^−1^) with FluoSpheres (Invitrogen) for four consecutive days on the same region. BrdU administration was done either intraperitoneally in PBS or via drinking water bottle (25 μg per g of body weight) throughout the duration of injections. For delivery of demethylase inhibitors, GSK J1 (#4593, TOCRIS), and JIB (#4972, TOCRIS) were dissolved in dimethylsulphoxide at 100 mM concentration. 500 μM of each were diluted in 100% acetone. Back skin hairs of CD1 × FVB wild-type mice were shaved and 100 μl of inhibitors (in acetone) were topically applied to the shaved region, twice per day for 1 or 2 weeks (see Fig. 7a for experimental scheme). Control mice were treated with acetone containing the same amount of dimethylsulphoxide.

### Fluorescence-activated cell sorting

For all FACS experiments, we used a protocol that has previously been described^46^. Briefly, mouse back and belly skin cells were isolated using trypsin digestion and filtered through 70 and 40 μm cell strainers (BD Biosciences) to obtain single-cell suspensions. Cells were labelled with α-CD34-Biotin (1:50, eBiosciences), α-Streptavidin-APC (1:100, BD Pharmingen) and α-α6-integrin (1:40, CD49f, BD Pharmingen). Propidium iodide (1:1,250–1:2,500 of 1 mg ml^−1^ stock, Sigma) was used to label the dead cells. BD FACSAria in the Flow Cytometry Core at Cornell University was used for cell sorting.

### Cell culture

For growth factor removal, mixed strain (129 × BL6) of wild-type newborn mouse skin was used as described to derive primary keratinocytes, which were expanded and used for experiments (between passages 4–6)^47^. Cells were first grown to confluency. Then, cells were washed once with PBS and added serum-free media containing either recombinant BMP4 (R&D Systems) between 1 and 2 nM or its carrier (4 mM HCl in 0.1%BSA) only. Cells incubated for 6 and 12 h were harvested for RNA extractions.

### Immunofluorescence staining

Optimal cutting temperature (OCT) compound-embedded frozen skin blocks were cryosectioned, fixed, immuno-blocked and incubated with antibodies. Antibodies and dilutions used for staining are H3K4me3 (1:500, ActiveMotif), H3K9me3 (1:500, Millipore and Abcam), H3K27me3 (1:500–1:1,000, Millipore), BrdU (1:300, Abcam), CD34 (1:150, BD Pharmingen) and Ki67 (1:200, Novocastra). A fluorescence light microscope (Nikon) equipped with a charge-coupled device 12-bit digital camera (Retiga EXi; QImaging) was used to analyse all stained slides.

### Western blot

For histone protein detection, we either FACS-isolated bulge and non-bulge cells or extracted chromatin from whole skin snap frozen in liquid N_2_. From FACS-isolated bulge and non-bulge cells, cells were first incubated in Triton Extraction Buffer (PBS containing 0.5% Triton × 100 (volume per volume), 2 mM phenylmethylsulfonyl fluoride), followed by 0.2 N HCl incubation. H3K9me3 (1:500–1: 3,000; Millipore) and H3 (1:3,000–1: 10,000; Abcam) antibodies were used for immunoblotting. For whole skin chromatin extraction, tissue samples were first lysed with Buffer A (1 M HEPES-KOH, pH7.8, 3 M KCl, 1 M MgCl_2_, 1 M sucrose, glycerol and protease/phosphatase inhibitors), and the pellet after spin down was incubated with Buffer B (0.5 M EDTA, 0.5 M EGTA, and protease/phosphatase inhibitors). H3K4me3 (Active motif, 1:1,000), H3K9me3 (Abcam, 1:2,000), H3K27me3 (Upstate, 1:1,000), H3 (Abcam, 1:2,000) antibodies were used for immunoblotting. ImageJ (http://imagej.nih.gov/ij/) and AutoQuant (MediaCybernetics) were used for normalization and quantifications. Raw western blot scanned images are included in Supplementary Fig. 5.

### Quantitative real-time reverse transcriptase–PCR

A previously described method was used for RNA extraction and qRT–PCR^46^. Briefly, RNeasy Micro kit (Qiagen) was used to extract RNA from FACS-isolated cells. All RNA samples were quality checked with Bioanalyer (Bio-Rad) through Cornell Life Sciences Core Laboratories Center. cDNA synthesis was carried out using iScript (Bio-Rad) cDNA synthesis kit according to the manufacturer's instruction. Quantitative real-time PCR reaction was carried out using homebrew SYBR Green PCR buffer. iCycler (Bio-Rad) PCR machine was used to detect fluorescence. Primers used for qRT–PCR are listed in Supplementary Table 1.

### ChIP and ChIP-seq library preparation

For ChIP using keratinocytes, a previously described method was used^46^ with minor variations. 3–5 μg of H3 (Abcam), H3K4me3 (ActiveMotif), H3K9me3 (Millipore or Abcam), H3K27me3 (Millipore) and IgG (Cell Signaling) were used for immunoprecipitation. For ChIP using FACS-isolated cells, 15–20 mice of CD1 × FVB wild-type strain between PD22–25 (early anagen, anagen I–IIIa (ref. 48)) and PD38–44 (late catagen, catagen VII–VIII (ref. 48)) were used. The stages were checked by histological sections. ∼3–4 million bulge and non-bulge cell nuclei were briefly fixed (1%, 1–2 min). Cells were dounced between 30 and 40 times in Buffer A (300 mM sucrose, 2 mM Mg acetate, 3 mM CaCl_2_, 10 mM Tris-Cl, pH 8.0, 0.1% Triton X-100, 0.5 mM dithiothreitol (DTT)) and washed (25% glycerol, 5 mM Mg acetate, 50 mM Tris, pH 8.0, 0.1 mM EDTA, 5 mM DTT). Pellet was resuspended in MNase buffer (60 mM KCl, 15 mM NaCl, 15 mM Tris-HCl, pH 7.4, 0.5 mM DTT, 0.25 M sucrose, 1.0 mM CaCl_2_) and treated with microccoccal nuclease (MNase) (50U, Affymetrix; ∼12 min, room temperature) to generate mono-nucleosomal fragments. Isolated chromatin from each sorted cell population was split into four and immunoprecipitated with antibodies indicated above. Eluted ChIP DNAs were either used for sequencing library generation. Library preparation was done essentially as described^49^. Briefly, ChIP DNA was quantified using Quant-iT dsDNA HS reagent (Invitrogen). Nanograms of ChIP DNA for each sample were processed using following steps: (i) end-repair using End-It DNA End-Repair Kit (Epicentre Biotechnologies), (ii) addition of single A-base using Klenow Fragment (3′->5′ exo-; New England Biolabs) and dATP (Invitrogen), (iii) ligation of barcoded adapters to A-overhang (Enzymatics), (iv) gel purification using SYBR gold (Invitrogen) and low Tm agarose gel, (v) PCR enrichment (13–14 cycles) using Phusion Polymerase (New England Biolabs), (vi) PCR purify using AMPure kit (Agencourt), (vii) native acrylamide gel purification, (viii) phenol/chloroform/isoamylalchol extraction and (ix) ethanol precipitation. Purified DNA samples were measured in their quality and quantity using bioanalyser before sequencing.

### ChIP-seq data analysis

*Overall workflow* ChIP library of H3K4me3, H3K27me3, H3K9me3 and IgG from EA-HFSCs, LC-HFSCs, non-EA-HFSCs and non-LC-HFSCs were sequenced (single-end) using Illumina GAIIx (86 bp) or HiSeq2000 (100 bp) at the Cornell University Institute of Biotechnology. All sequenced reads were processed as following:

- Filtering of passed reads.
- Barcode split followed by de-barcoding multiplexed samples.
- Clipping of adapter sequence that is contaminated in the reads.
- Trimming low-quality ends. Based on the resulting read-length distribution reads were trimmed to a uniform length of 70 bp.
- Filtering out reads that have 5 (of 70) base pair or more with low quality score.
- Alignment to the mouse genome (mm9) using Bowtie2 (ref. 50).
- Filtering out low-quality aligned reads using bamtools.
- Peak calling of (vii) using MACS (Model-based Analysis of ChIP-seq)^24^.
- Complimentary peak calling of (vii) using HOMER^51^.
- Combining and merging all MACS peaks from (viii) into a customized non-overlapping annotation.
- Quantification of (vii) using HOMER or Cuffdiff^25^ on pre-annotated genomic location as well as on customized annotation from (x).
- Correlation analyses of ChIP-seq reads with gene expression changes.

#### Experimental replicates

Experimental replicates (independent pools of HFSCs/non-HFSCs were isolated from independent sets of mice for ChIP). The two experimental replicates showed a high Pearson correlation coefficient for all three marks (Supplementary Fig. 2a).

#### Peak calling

MACS peak detection showed near saturation of H3K4me3 in all populations analysed. However, the number of reads was not sufficient for H3K27me3 and H3K9me3 to be saturated by MACS. This is not uncommon as both histone marks are broadly enriched throughout the genome. Therefore, to get a better representation of the distribution and enriched regions, we pooled reads from experimental replicates and analysed them together subsequently. In the end, for each sample, there were between 10 and 40 million uniquely mapped reads (except for IgG, which were between 5 and 20 million reads).

Conditions used for MACS are listed below.

For H3K4me4: macs14—tsize=70—*P*-value=1e-5—keep-dup=2—nomodel—mfold=10,40.

For H3K27me3 and H3K9me3: macs14—tsize=70—*P*-value=1e-4—keep-dup=2—nomodel—mfold=5,40.

IgG for each population was used as control for all peak calling.

Conditions used for HOMER are listed below.

findPeaks -d <tag directory> -style histone -tbp 2 -o auto -i <IgG tag directory> -F 5

Interestingly, in both analyses, number of peaks called in LC-HFSCs was significantly less than in EA-HFSCs for all three histone modification marks (Fig. 2). This was not due to the smaller number of reads sequenced. In fact, more reads were used in the LC-HFSCs (∼30–40 million) than in the EA-HFSCs (∼10–30 million). These suggest that the reads in LC-HFSCs are either not significantly enriched relative to the background or are more spread out throughout the genome.

#### Quantification of ChIP-seq reads

The primary purpose of peak calling is to identify a specific region of enrichment over the background. The peak calling methods generally calculate the fold enrichment relative to background in a given window. Thus, these values are not necessarily quantitative and comparable across different genomic regions and more importantly, are not directly comparable between two samples. To more quantitatively measure and directly compare enrichment among samples, we developed an alternative ChIP-seq analyses method.

We first called peaks using MACS for all marks in all populations using methods described in the section Peak calling. The peaks were merged, sorted and overlapping regions were combined to generate one customized annotation file containing all genomic regions displaying peaks in any of the populations analysed. This file was used to extract either the tag density (HOMER) or FPKM (Cuffdiff) values, using an RNAseq analysis pipeline. The values extracted are quantitative and are comparable across samples since both values are normalized to the overall number of mapped reads.

Using this approach, we identified 201,178 loci that cover ∼330 Mb (∼12%) of the total genome. These regions included various sites of TSS/promoters, exons, introns, intergenic regions, CpGs, untranslated regions, non-coding RNAs and multiple repeat sequences. After subtracting the normalized IgG signal at each locus, cluster analysis was performed to generate a heat map (Fig. 3). We also used other pre-annotated files available in HOMER (such as TSS) to quantify and compare the reads at or around those sites. Normalized data were used in all ChIP-seq analyses done in this study except for when comparing normalized to the pre-normalized samples (Supplementary Fig. 3).

#### Correlation of histone marks and gene expression

For gene expression, previous microarray data sets were used. Briefly, K5tTA × pTRE-H2B-GFP transgenic mice were doxy-induced from PD18–21 (ref. 16), PD22–25 (ref. 16) or PD21–42 (unpublished T. Tumbar and Y. V. Zhang). Cells from different H2B-GFP division peaks were sorted from the bulge and non-bulge. For correlation analyses done in this paper, signals from multiple peaks were combined based on the fraction of cells in each population, in order to reconstitute a single bulge signal for PD25 and PD42. Next, the reconstituted signals were divided between PD42 and PD25 bulges to get the fold changes of all genes. The fold changes were converted into log_2_ scale. For correlation between LC-HFSCs and non-LC-HFSCs, the PD42 non-bulge (CD34-) signals and the reconstituted PD42 bulge signals were compared. The reconstituted array signals are included in Supplementary Data 1.

ChIP-seq reads from EA-HFSCs, LC-HFSCs and non-LC-HFSCs were quantified at 5 kb spanning the TSSs (HOMER). Quantified reads were IgG normalized. The signals were divided between EA-HFSCs and LC-HFSCs, or between LC-HFSCs and non-LC-HFSCs to get the fold changes of all genes. The fold changes were converted into log_2_ scale. These values were matched with array signal for each gene. The data were then converted into density and plotted using R.

## Additional information

**Accession codes:** ChIP-seq data have been deposited in the NCBI Gene Expression Omnibus (GEO) database under accession code GSE78749.

**How to cite this article:** Lee, J. *et al*. Signalling couples hair follicle stem cell quiescence with reduced histone H3 K4/K9/K27me3 for proper tissue homeostasis. *Nat. Commun.* 7:11278 doi: 10.1038/ncomms11278 (2016).

## Supplementary Material

Supplementary Information Supplementary Figures 1-5, Supplementary Table 1 and Supplementary Reference

Supplementary Data 1 Reconstituted microarray signal from HFSCs in catagen (PD42)

Supplementary Data 2 List of subsets of genes in their correlation of changes in histone marks and gene expression between EA-HFSCs and LC-HFSCs

## References

[b1] Onder T. T. . Chromatin-modifying enzymes as modulators of reprogramming. Nature 483, 598–602 (2012). 22388813 10.1038/nature10953 PMC3501145

[b2] Hawkins R. D. . Distinct epigenomic landscapes of pluripotent and lineage-committed human cells. Cell Stem Cell 6, 479–491 (2010). 20452322 10.1016/j.stem.2010.03.018 PMC2867844

[b3] Mattout A. & Meshorer E. Chromatin plasticity and genome organization in pluripotent embryonic stem cells. Curr. Opin. Cell Biol. 22, 334–341 (2010). 20226651 10.1016/j.ceb.2010.02.001

[b4] Hubner M. R., Eckersley-Maslin M. A. & Spector D. L. Chromatin organization and transcriptional regulation. Curr. Opin. Genet. Dev. 23, 89–95 (2013). 23270812 10.1016/j.gde.2012.11.006 PMC3612554

[b5] Cavalli G. & Misteli T. Functional implications of genome topology. Nature Struct. Mol. Biol. 20, 290–299 (2013). 23463314 10.1038/nsmb.2474 PMC6320674

[b6] Blanpain C. & Fuchs E. Stem cell plasticity. Plasticity of epithelial stem cells in tissue regeneration. Science 344, 1242281 (2014). 24926024 10.1126/science.1242281 PMC4523269

[b7] Adam R. C. . Pioneer factors govern super-enhancer dynamics in stem cell plasticity and lineage choice. Nature 521, 366–370 (2015). 25799994 10.1038/nature14289 PMC4482136

[b8] Boyer L. A. . Polycomb complexes repress developmental regulators in murine embryonic stem cells. Nature 441, 349–353 (2006). 16625203 10.1038/nature04733

[b9] Loh Y. H., Zhang W., Chen X., George J. & Ng H. H. Jmjd1a and Jmjd2c histone H3 Lys 9 demethylases regulate self-renewal in embryonic stem cells. Genes Dev. 21, 2545–2557 (2007). 17938240 10.1101/gad.1588207 PMC2000320

[b10] Chen J. . H3K9 methylation is a barrier during somatic cell reprogramming into iPSCs. Nature Genet. 45, 34–42 (2013). 23202127 10.1038/ng.2491

[b11] Baxter J. . Histone hypomethylation is an indicator of epigenetic plasticity in quiescent lymphocytes. EMBO J. 23, 4462–4472 (2004). 15510223 10.1038/sj.emboj.7600414 PMC526455

[b12] Liu L. . Chromatin modifications as determinants of muscle stem cell quiescence and chronological aging. Cell Rep. 4, 189–204 (2013). 23810552 10.1016/j.celrep.2013.05.043 PMC4103025

[b13] Fuchs E. The tortoise and the hair: slow-cycling cells in the stem cell race. Cell 137, 811–819 (2009). 19490891 10.1016/j.cell.2009.05.002 PMC2716122

[b14] Lee J. & Tumbar T. Hairy tale of signaling in hair follicle development and cycling. Sem. Cell Dev. Biol. 23, 906–916 (2012). 10.1016/j.semcdb.2012.08.003 PMC3496046 22939761

[b15] Blanpain C. & Fuchs E. Epidermal homeostasis: a balancing act of stem cells in the skin. Nat. Rev. Mol. Cell Biol. 10, 207–217 (2009). 19209183 10.1038/nrm2636 PMC2760218

[b16] Zhang Y. V., Cheong J., Ciapurin N., McDermitt D. J. & Tumbar T. Distinct self-renewal and differentiation phases in the niche of infrequently dividing hair follicle stem cells. Cell Stem Cell 5, 267–278 (2009). 19664980 10.1016/j.stem.2009.06.004 PMC2756832

[b17] Rompolas P. . Live imaging of stem cell and progeny behaviour in physiological hair-follicle regeneration. Nature 487, 496–499 (2012). 22763436 10.1038/nature11218 PMC3772651

[b18] Greco V. . A two-step mechanism for stem cell activation during hair regeneration. Cell Stem Cell 4, 155–169 (2009). 19200804 10.1016/j.stem.2008.12.009 PMC2668200

[b19] Lee S. E. . High Runx1 levels promote a reversible, more-differentiated cell state in hair-follicle stem cells during quiescence. Cell Rep. 6, 499–513 (2014). 24462289 10.1016/j.celrep.2013.12.039 PMC4052453

[b20] Sada A. & Tumbar T. New insights into mechanisms of stem cell daughter fate determination in regenerative tissues. Int. Rev. Cell Mol. Biol. 300, 1–50 (2013). 23273858 10.1016/B978-0-12-405210-9.00001-1 PMC5788169

[b21] Wabik A. & Jones P. H. Switching roles: the functional plasticity of adult tissue stem cells. EMBO J. 34, 1164–1179 (2015). 25812989 10.15252/embj.201490386 PMC4426478

[b22] Jenuwein T. & Allis C. D. Translating the histone code. Science 293, 1074–1080 (2001). 11498575 10.1126/science.1063127

[b23] Lien W. H. . Genome-wide maps of histone modifications unwind *in vivo* chromatin states of the hair follicle lineage. Cell Stem Cell 9, 219–232 (2011). 21885018 10.1016/j.stem.2011.07.015 PMC3166618

[b24] Zhang Y. . Model-based analysis of ChIP-Seq (MACS). Genome Biol. 9, R137 (2008). 18798982 10.1186/gb-2008-9-9-r137 PMC2592715

[b25] Trapnell C. . Differential analysis of gene regulation at transcript resolution with RNA-seq. Nature Biotechnol. 31, 46–53 (2013). 23222703 10.1038/nbt.2450 PMC3869392

[b26] Marmorstein R. & Trievel R. C. Histone modifying enzymes: structures, mechanisms, and specificities. Biochim. Biophys. Acta 1789, 58–68 (2009). 18722564 10.1016/j.bbagrm.2008.07.009 PMC4059211

[b27] Tumbar T. . Defining the epithelial stem cell niche in skin. Science 303, 359–363 (2004). 14671312 10.1126/science.1092436 PMC2405920

[b28] Wang L. . A small molecule modulates Jumonji histone demethylase activity and selectively inhibits cancer growth. Nat. Commun. 4, 2035 (2013). 23792809 10.1038/ncomms3035 PMC3724450

[b29] Kruidenier L. . A selective jumonji H3K27 demethylase inhibitor modulates the proinflammatory macrophage response. Nature 488, 404–408 (2012). 22842901 10.1038/nature11262 PMC4691848

[b30] Waghmare S. K. . Quantitative proliferation dynamics and random chromosome segregation of hair follicle stem cells. EMBO J. 27, 1309–1320 (2008). 18401343 10.1038/emboj.2008.72 PMC2374848

[b31] Plikus M. V. . Cyclic dermal BMP signalling regulates stem cell activation during hair regeneration. Nature 451, 340–344 (2008). 18202659 10.1038/nature06457 PMC2696201

[b32] Cobb B. S. . Targeting of Ikaros to pericentromeric heterochromatin by direct DNA binding. Genes Dev. 14, 2146–2160 (2000). 10970879 10.1101/gad.816400 PMC316893

[b33] Zentner G. E. & Henikoff S. Regulation of nucleosome dynamics by histone modifications. Nat. Struc. Mol. Biol. 20, 259–266 (2013). 10.1038/nsmb.2470 23463310

[b34] Rompolas P., Mesa K. R. & Greco V. Spatial organization within a niche as a determinant of stem-cell fate. Nature 502, 513–518 (2013). 24097351 10.1038/nature12602 PMC3895444

[b35] Ito M., Kizawa K., Hamada K. & Cotsarelis G. Hair follicle stem cells in the lower bulge form the secondary germ, a biochemically distinct but functionally equivalent progenitor cell population, at the termination of catagen. Differentiation 72, 548–557 (2004). 15617565 10.1111/j.1432-0436.2004.07209008.x

[b36] Zhang Y. V., White B. S., Shalloway D. I. & Tumbar T. Stem cell dynamics in mouse hair follicles: a story from cell division counting and single cell lineage tracing. Cell Cycle 9, 1504–1510 (2010). 20372093 10.4161/cc.9.8.11252 PMC3096686

[b37] Sander J. D. & Joung J. K. CRISPR-Cas systems for editing, regulating and targeting genomes. Nature Biotechnol. 32, 347–355 (2014). 24584096 10.1038/nbt.2842 PMC4022601

[b38] Herrera-Merchan A. . Ectopic expression of the histone methyltransferase Ezh2 in haematopoietic stem cells causes myeloproliferative disease. Nat. Commun. 3, 623 (2012). 22233633 10.1038/ncomms1623

[b39] Kandyba E. . Competitive balance of intrabulge BMP/Wnt signaling reveals a robust gene network ruling stem cell homeostasis and cyclic activation. Proc. Natl Acad. Sci. USA 110, 1351–1356 (2013). 23292934 10.1073/pnas.1121312110 PMC3557042

[b40] Genander M. . BMP signaling and its pSMAD1/5 target genes differentially regulate hair follicle stem cell lineages. Cell Stem Cell 15, 619–633 (2014). 25312496 10.1016/j.stem.2014.09.009 PMC4276600

[b41] Lien W. H. . *In vivo* transcriptional governance of hair follicle stem cells by canonical Wnt regulators. Nat. Cell Biol. 16, 179–190 (2014). 24463605 10.1038/ncb2903 PMC3984009

[b42] Sen G. L., Webster D. E., Barragan D. I., Chang H. Y. & Khavari P. A. Control of differentiation in a self-renewing mammalian tissue by the histone demethylase JMJD3. Genes Dev. 22, 1865–1870 (2008). 18628393 10.1101/gad.1673508 PMC2492733

[b43] Simons B. D. & Clevers H. Strategies for homeostatic stem cell self-renewal in adult tissues. Cell 145, 851–862 (2011). 21663791 10.1016/j.cell.2011.05.033

[b44] Perez-Lluch S. . Absence of canonical marks of active chromatin in developmentally regulated genes. Nature Genet. 47, 1158–1167 (2015). 26280901 10.1038/ng.3381 PMC4625605

[b45] Oshimori N. & Fuchs E. Paracrine TGF-beta signaling counterbalances BMP-mediated repression in hair follicle stem cell activation. Cell Stem Cell 10, 63–75 (2012). 22226356 10.1016/j.stem.2011.11.005 PMC3349223

[b46] Lee J. . Runx1 and p21 synergistically limit the extent of hair follicle stem cell quiescence *in vivo*. Proc. Natl Acad. Sci. USA 110, 4634–4639 (2013). 23487742 10.1073/pnas.1213015110 PMC3606971

[b47] Osorio K. M., Lilja K. C. & Tumbar T. Runx1 modulates adult hair follicle stem cell emergence and maintenance from distinct embryonic skin compartments. J. Cell Biol. 193, 235–250 (2011). 21464233 10.1083/jcb.201006068 PMC3082184

[b48] Muller-Rover S. . A comprehensive guide for the accurate classification of murine hair follicles in distinct hair cycle stages. J. Invest. Dermatol. 117, 3–15 (2001). 11442744 10.1046/j.0022-202x.2001.01377.x

[b49] Guertin M. J. & Lis J. T. Chromatin landscape dictates HSF binding to target DNA elements. PLoS Genet. 6, e1001114 (2010). 20844575 10.1371/journal.pgen.1001114 PMC2936546

[b50] Langmead B., Trapnell C., Pop M. & Salzberg S. L. Ultrafast and memory-efficient alignment of short DNA sequences to the human genome. Genome Biol. 10, R25 (2009). 19261174 10.1186/gb-2009-10-3-r25 PMC2690996

[b51] Heinz S. . Simple combinations of lineage-determining transcription factors prime cis-regulatory elements required for macrophage and B cell identities. Mol. Cell 38, 576–589 (2010). 20513432 10.1016/j.molcel.2010.05.004 PMC2898526

