# Supplementary material for: Signalling couples hair follicle stem cell quiescence with reduced histone H3 K4/K9/K27me3 for proper tissue homeostasis
Source: Nat Commun. 2016 Apr 15;7:11278. doi: 10.1038/ncomms11278 (PMC4835553; doi:10.1038/ncomms11278)
Supplement: Supplementary Information — Supplementary Figures 1-5, Supplementary Table 1 and Supplementary Reference [file ncomms11278-s1.pdf]

Supplementary Figure 1.

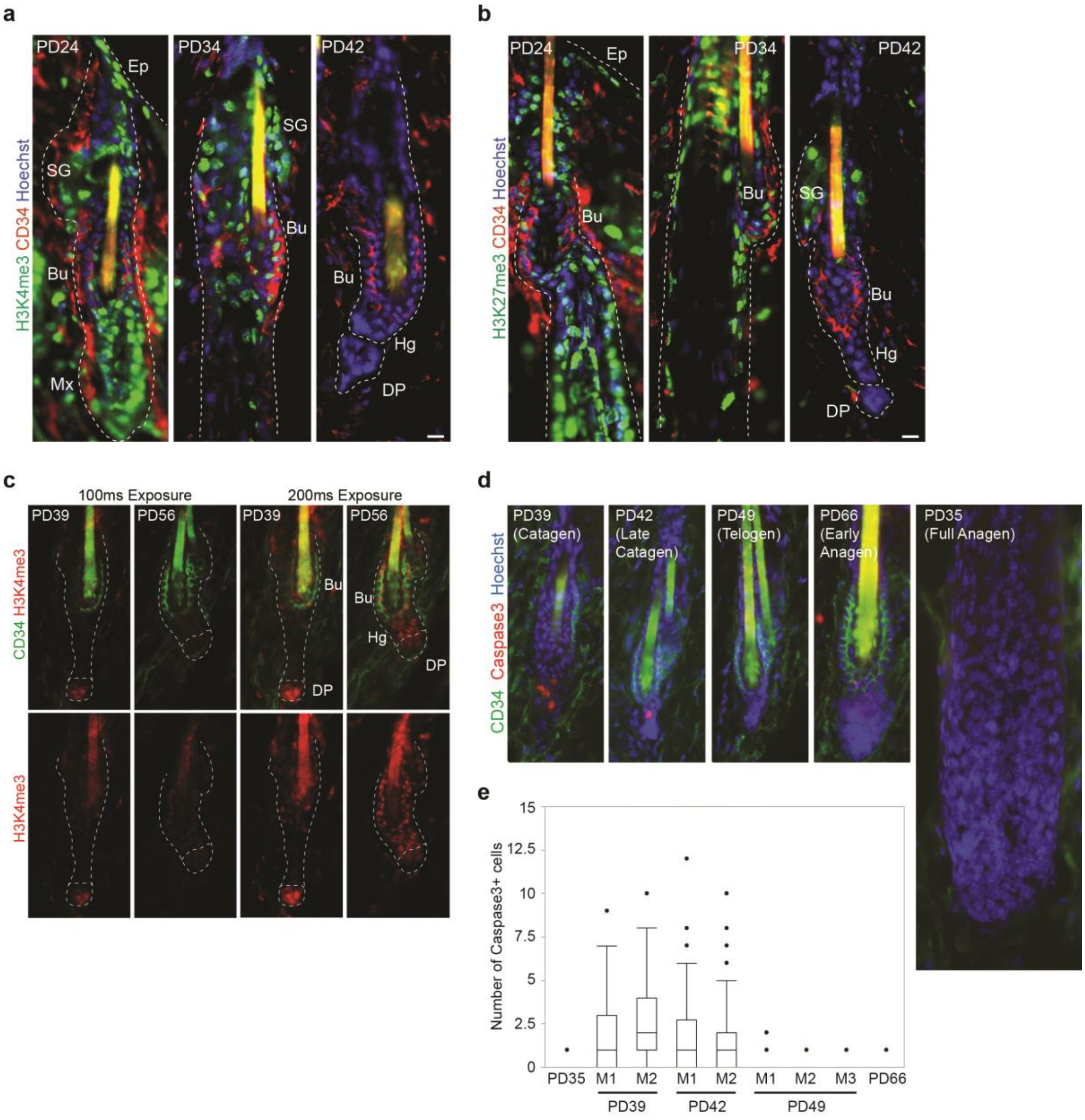

Supplementary Figure 1. Quiescent (catagen) HFSCs display globally lower levels of histone H3 K4me3, K9me3, K27me3 than proliferative (early anagen) HFSCs and differentiated cells. (a-b) Immunofluorescence (IF) staining of (a) H3K4me3 and (b) H3K27me3 (green) overlaid with CD34 (red) at hair cycle stages (PD24, 34, and 42) indicated (Ep: Epidermis; Mx; Matrix; Bu: Bulge; Hg; Hair germ; DP: Dermal Papillae). Notice the reduction in methyl marks in PD42, both in the bulge and in the dermis. Scale bars, 10µm. (c) Representative IF image of H3K4me3 from PD39 (catagen) and PD56 (early anagen) taken at different exposures. Note that in catagen, the marks are not completely erased but rather the levels are reduced. (d-e) IF staining with an apoptotic marker Caspase-3 (d) and its quantifications (e) reveal that the stage where the lowest histone mark levels are observed coincides with catagen. (e) A box whisker plot of number of caspase + cells across different hair cycle stages. The box includes the middle 50% (between 25% and 75%) of the sample. The line inside the box indicates the mean value. The whiskers indicate the maximum excluding outliers (defined as above/below 1.5x interquartile range). Note that this stage is different from previous ChIP-seq study done by *Lien et al*[1].

Supplementary Figure 2.

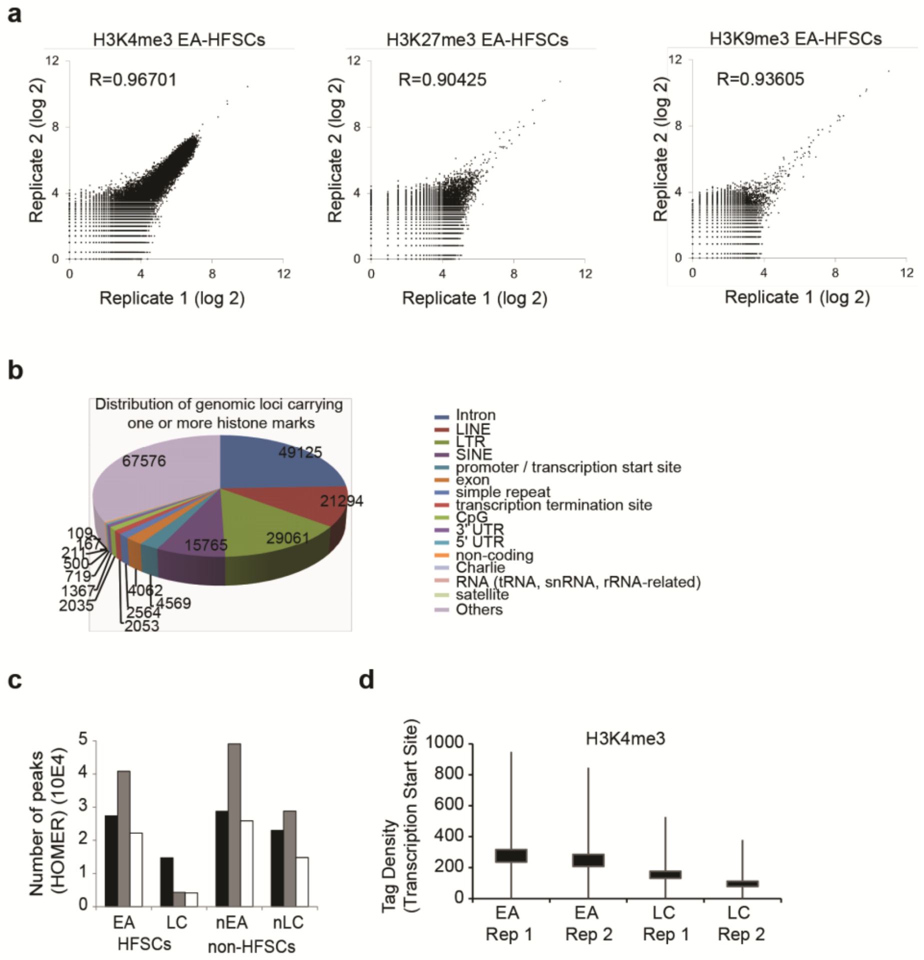

Supplementary Figure 2. ChIP-seq data analysis and comparison with the study *Lien et al*.[1] shows similar, yet distinct genome-wide enrichment. (a) Pearson correlation analysis of each histone mark between the replicates. (b) Pie chart of all genomic regions covered by MACS peaks from three marks in all four populations. (c) Total number of peaks called using another peak caller (HOMER) also show dramatic reduction in the total number of peaks called in LC-HFSCs. (d) Box whisker plot of tag density of H3K4me3 in EA-HFSCs and LC-HFSCs at TSS (normalized to 5000bp in 10 million of total mapped reads). Although the number of peaks detected between EA-HFSCs and LC-HFSCs are similar, there is a global reduction of peak signals in LC-HFSCs.

Supplementary Figure 3.

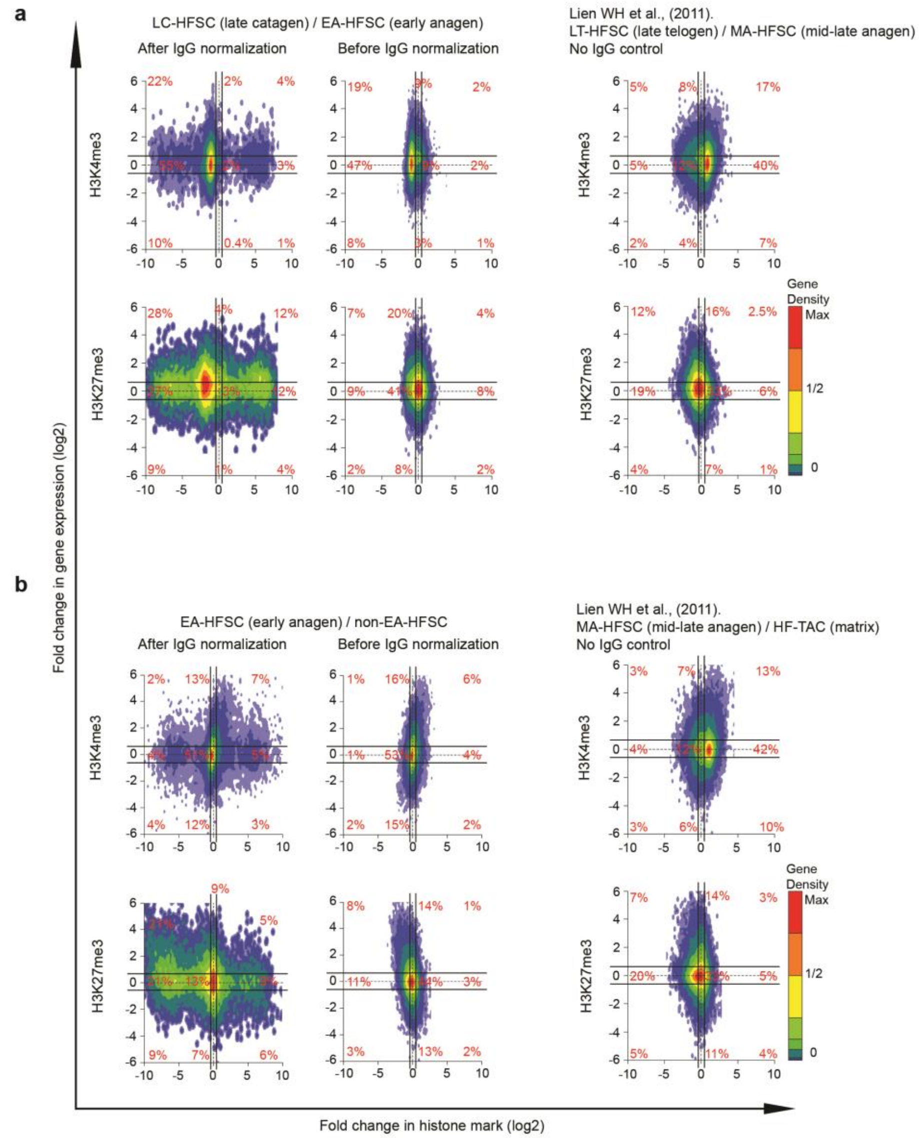

Supplementary Figure 3. Correlation of changes in histone marks and gene expression between *Lien et al.*[1] and this study. Density plot of histone marks and gene expression changes between EA-HFSCs and LC-HFSCs (a) and EA-HFSCs and non-EA-HFSCs (b). Before (middle panels) and after (left panels)) IgG normalization is shown. Note significant difference introduced by IgG normalization in ChIP-seq data, which cannot be accounted for using input DNA alone as control. IgG control accounts for background binding of antibodies to specific chromatin regions, to account for secondary effects on ChIP results of antibody penetration to the DNA (due to potential differences in chromatin compaction). (Right panels) ChIP-seq reads from *Lien et al*.[1] were re-analyzed using our quantitative methods, but IgG control was not available from this study. HFSCs in this previous study were sorted at Mid Anagen (MA-HFSCs, PD28-30) (ours were at early anagen) and at Late Telogen (LT-HFSCs, PD52-58) (ours are sorted at late catagen). Panels in (a) show density plot of correlation between MA-HFSCs and LT-HFSCs and between MA-HFSCs and HF-TAC (Hair Follicle-Transit Amplifying Cells, sorted matrix cells in *Lien et al*. (b). Note similar results obtained for H3K27me3 from our and data sets of *Lien et al*.[1], and the accentuation of H3K27me3 level changes at transition to quiescence by using IgG as control. H3K4me3 appears globally increased in the data set of *Lien et al*.[1] in telogen (LT-HFSCs) than MA-HFSCs and HFTACs. This may be attributed to the difference in hair cycle stages analyzed.

Supplementary Figure 4.

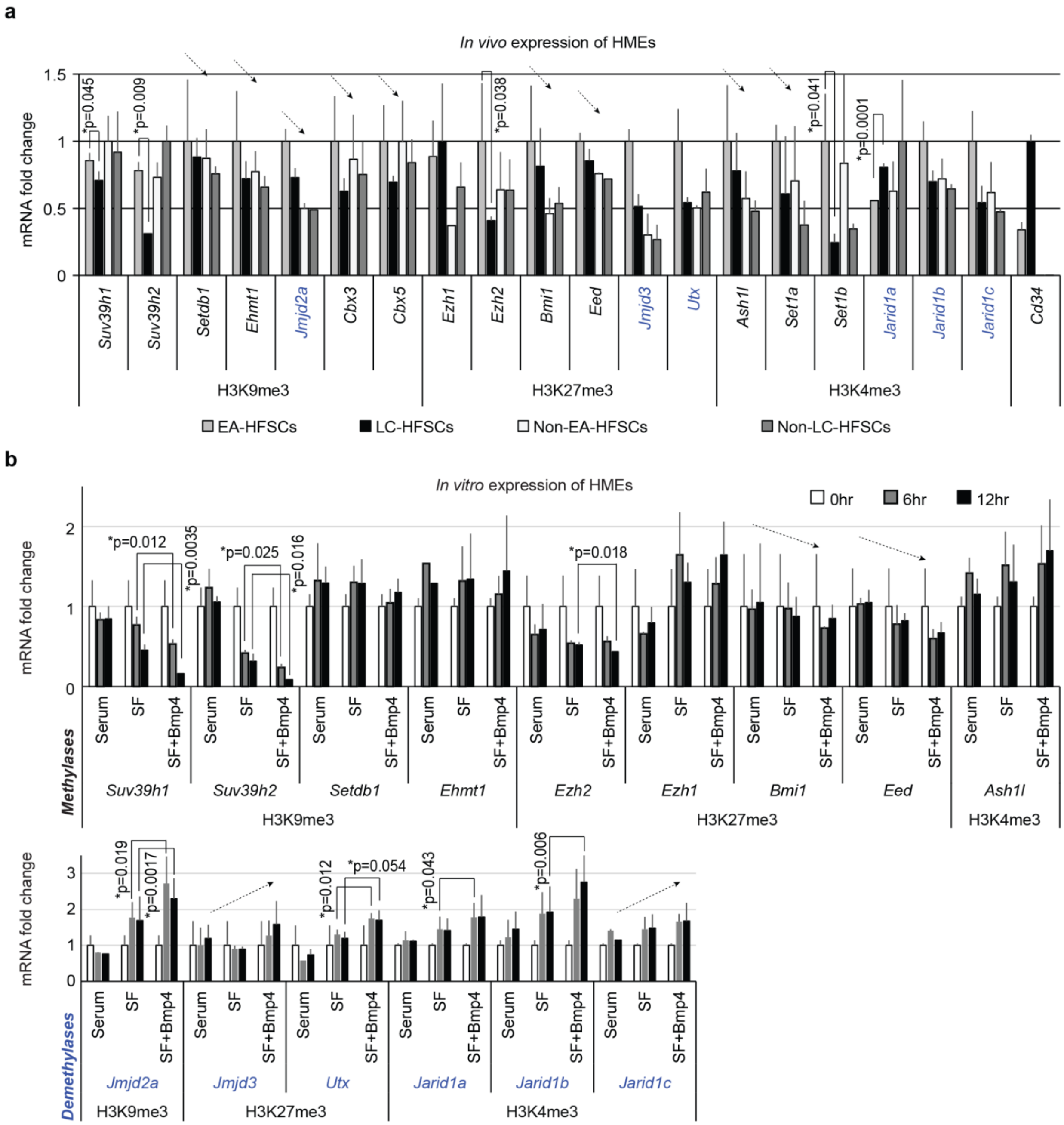

Supplementary Figure 4. Expression of histone modifying enzymes (HMEs) *in vivo* and *in vitro*. Full list of chromatin genes associated with methylation and demethylation tested by qRT-PCR (a) *in vivo* and (b) *in vitro*. Methylases are marked in black and demethylases in blue. (a) For each gene, expression of the highest expressing population of four sorted samples (EA-HFSCs, LC-HFSCs, non-EA-HFSCs, and non-LC-HFSCs) was normalized to 1. Dotted arrows are to show the trend of down-regulation between EA-HFSCs and LC-HFSCs for genes with $p>0.05$. (b) For each gene, expression in 0hr was normalized to 1. Dotted lines also show trends of genes that display similar up- or down-regulation but did not meet statistical significance of $p<0.05$ by Student's t-test.

Supplementary Figure 5

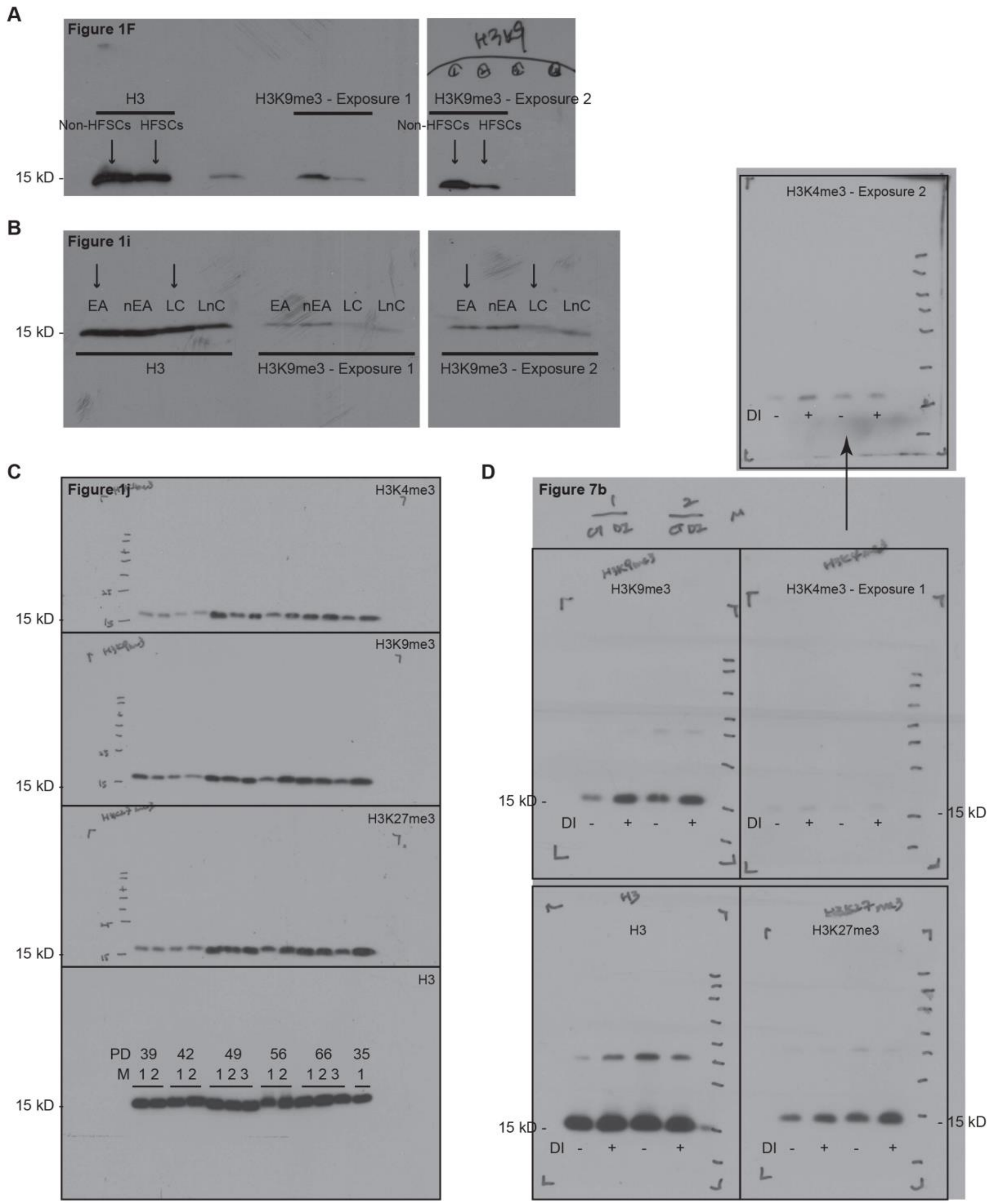

Supplementary Figure 5. Raw western blots used in this study. (a) Original blot of Fig. 1f. Arrows indicate the actual bands used for the figure. 'Exposure 2' was developed longer than 'Exposure 1' to obtain brighter band intensities. (b) Original blot of Fig. 1i. Arrows indicate the actual bands used for the figure. EA: Early Anagen HFSCs; nEA: Early Anagen Non-HFSCs; LC: Late Catagen HFSCs; nLC: Late Catagen Non-HFSCs. (c) Original blot of Fig. 1j. PD: Postnatal Days; M: Mouse (d) Original blots of Fig. 7b. 'Exposure 2' was developed longer than 'Exposure 1' to obtain brighter band intensities. DI: Demethylase Inhibitor.

Supplementary Table 1. List of primers used.

Gene/Region	Forward	Reverse
Suv39h1	GCTCTGCCTCCTCTGAGGTAA	TCTCTGCATCTTCCGCACTA
Suv39h2	CTGCCCAGGATAGCATTGTTC	CAAGTCTCGGCTCCACATTTAC
Ezh2	AGTGACTTGGATTTTCCAGCAC	TCACCATGCACTTTTCCATCAT
Jarid1a	TTTATCGGGCGCATCCGGCC	TCATTGCCTCAAGTTCATTCAGGCG
Jmjd2a	GCGACCCTTGGTCTTCTTATT	GGAGAGCCGAGTGTGAAGAA
Utx	CGGGCGGACAAAAGAAGAAC	CATAGACTTGCATCAGATCCTCC
Setdb1	TGCAGCGTTTGTTACACTCA	CCAGCTAACTATCCAGGCCA
Ehmt1	TGGCCACCACAAAGTCCCAGACA	GCTTTCTCGCCCTTGGCGCC
Cbx3	ACTGGACCGTCGTGTAGTGAA	GCCCCTTGGTTTGTCAGCA
Cbx5	GACAGGCGCATGGTTAAGG	CCTGGGCTTATTGTTTTCACCC
Ezh1	TCCAGTATGTGATGCATTTGG	TGCGTCTCAGGATGGAGG
Bmi1	TGATTCTGGTTGTTCGATGC	TGGCTCGCATTCATTTTATG
Jmjd3	TGAAGAACGTCAAGTCCATTGTG	TCCCGCTGTACCTGACAGT
Ash1l	AAGTGCATGGTGTGGCAGCACTG	CTGGGCATAGTGGGGCCTAGGG
Jarid1b	GAAGAGTTCGCGGACCCCTTCG	GTGTTTGGGCCTCCAGCTCATTCAG
Jarid1c	CACTGTGGTAAGCACGAGGA	CTCGTCACCCTCATGAATCC
CD34	AAGGCTGGGTGAAGACCCTTA	TGAATGGCCGTTTCTGGAAGT

Supplementary References

1 Lien, W. H. *et al.* Genome-wide maps of histone modifications unwind in vivo chromatin states of the hair follicle lineage. *Cell stem cell* **9**, 219-232, doi:10.1016/j.stem.2011.07.015 (2011).
